# A ‘synthetic-sickness’ screen for senescence re-engagement targets in mutant cancer backgrounds

**DOI:** 10.1371/journal.pgen.1006942

**Published:** 2017-08-14

**Authors:** Claire J. Cairney, Lauren S. Godwin, Alan E. Bilsland, Sharon Burns, Katrina H. Stevenson, Lynn McGarry, John Revie, Jon D. Moore, Ceri M. Wiggins, Rebecca S. Collinson, Clare Mudd, Elpida Tsonou, Mahito Sadaie, Dorothy C. Bennett, Masashi Narita, Christopher J. Torrance, W. Nicol Keith

**Affiliations:** 1 Institute of Cancer Sciences, Wolfson Wohl Cancer Research Centre, University of Glasgow, Glasgow, United Kingdom; 2 Molecular and Clinical Sciences Research Institute, St. George's, University of London, London, United Kingdom; 3 RNAi Screening Facility, Cancer Research UK Beatson Institute, Glasgow, United Kingdom; 4 Horizon Discovery Ltd, Cambridge Research Park, Waterbeach, Cambridge, United Kingdom; 5 Cancer Research UK Cambridge Institute, University of Cambridge, Cambridge, United Kingdom; 6 PhoreMost Ltd, Baraham Research Campus, Cambridge, United Kingdom; St Jude Children's Research Hospital, UNITED STATES

## Abstract

Senescence is a universal barrier to immortalisation and tumorigenesis. As such, interest in the use of senescence-induction in a therapeutic context has been gaining momentum in the past few years; however, senescence and immortalisation remain underserved areas for drug discovery owing to a lack of robust senescence inducing agents and an incomplete understanding of the signalling events underlying this complex process. In order to address this issue we undertook a large-scale morphological siRNA screen for inducers of senescence phenotypes in the human melanoma cell line A375P. Following rescreen and validation in a second cancer cell line, HCT116 colorectal carcinoma, a panel of 16 of the most robust hits were selected for further validation based on significance and the potential to be targeted by drug-like molecules. Using secondary assays for detection of senescence biomarkers p21, 53BP1 and senescence associated beta-galactosidase (SAβGal) in a panel of HCT116 cell lines carrying cancer-relevant mutations, we show that partial senescence phenotypes can be induced to varying degrees in a context dependent manner, even in the absence of p21 or p53 expression. However, proliferation arrest varied among genetic backgrounds with predominantly toxic effects in p21 null cells, while cells lacking PI3K mutation failed to arrest. Furthermore, we show that the oncogene ECT2 induces partial senescence phenotypes in all mutant backgrounds tested, demonstrating a dependence on activating *KRAS*^*G13D*^ for growth suppression and a complete senescence response. These results suggest a potential mechanism to target mutant KRAS signalling through ECT2 in cancers that are reliant on activating KRAS mutations and remain refractory to current treatments.

## Author summary

Cellular senescence is an irreversible arrest of cell proliferation. Senescence is understood to be a universal barrier that all cancers must overcome during their evolution. Developing ways to induce senescence in cancer cells is therefore an attractive strategy to identify targets for cancer therapy. However, a lack of understanding of this complex process has meant that little progress has been made in translating senescence induction into the clinic. Here we describe the identification and validation of a panel of inducers of senescence phenotypes from a large-scale siRNA screen. We show that the senescence response can be induced to varying degrees in genetic backgrounds mimicking common cancer mutations, allowing for a prioritisation of approaches. We also show that partial senescence responses can be triggered in cancer cells in the absence of genes considered by some to be essential for a senescence response. However, distinct differences in proliferation arrest are observed different backgrounds. These results advance our understanding of the complexity of senescence biology. This study has identified several potential targets for drug discovery. Interestingly, our results show the potential for therapeutic intervention in some backgrounds which are so far refractory to current treatments.

## Introduction

Cellular senescence, often described as the irreversible arrest of cell proliferation, can be induced by a variety of signals [1, 2]. It is a complex phenotype, consisting of various effector mechanisms including the DNA damage response (DDR), chromatin modification, autophagy and the senescence associated secretory phenotype (SASP) [3, 4]. Understanding collective control of these mechanisms is a priority [5] and screening approaches might help to deconvolute senescence pathways. However, the senescence response involves the expression of many biomarkers linked to these effector mechanisms, including cell cycle inhibitors such as p16 and p21, components of the SASP [6] and in some contexts senescence-associated heterochromatic foci (SAHF), which are associated with gene repression [7]. There are also some key morphological changes that occur, including a large flattened morphology, enlarged nucleus and expression of senescence associated beta-galactosidase (SAβGal). Expression of these biomarkers is context dependent and few specifically define senescence, therefore various markers should be investigated in parallel to confirm the senescent state [3, 6, 8].

Senescence markers and effectors are evident in various premalignant tissues *in vivo*, consistent with a role for senescence as a universal barrier to tumorigenesis. In mouse models of *KRAS*^*V12*^-dependent lung adenocarcinoma and pancreatic ductal carcinoma, p16 and SAβGal were detected in premalignant lesions, but not in the malignant disease [9]. Similarly, oncogenic *BRAF*^*V600E*^ overexpression can induce senescence in cultured melanocytes; human nevi (moles), which often carry *BRAF*^*V600E*^, express many senescence markers and much evidence supports a role for senescence in maintaining their arrested state and preventing progression to melanoma [10, 11].

Senescence signalling also occurs in advanced disease [4] and in response to standard chemo- or radiotherapy [12], where it may aid therapeutic activity of these agents by acting as a fail-safe mechanism in cases where pro-apoptotic signalling is defective [13]. Furthermore, induction of the SASP by cancer therapeutics may also induce a tumour-directed immune response owing to the release of inflammatory cytokines. Consistent with this, the re-expression of p53 in a p53-deficient mouse model of liver carcinoma expressing oncogenic *Hras*^*V12*^ resulted in senescence, rather than apoptosis, and tumour regression by immune clearance [14]. However, in some contexts SASP components could promote cell proliferation and tumorigenesis [15].

Senescence induction is an attractive concept in cancer research, and the idea of modulating the senescence response for therapeutic benefit, either to enhance current treatments or as a tumour suppressive therapy in its own right, has been gaining momentum over recent years. However, cellular senescence and immortality remain underserved areas for drug discovery owing to a lack of senescence-inducing agents and an incomplete knowledge of the complexity of the underlying signalling events. To address the first issue, we recently reported identification of a novel compound, CB-20903630, through a “target agnostic” virtual screen. The compound selectively induces a variety of senescence associated phenotypes and G1 blockade in cancer cells [16]. However, given the diversity of senescence triggers, it seems clear that engagement of the response is under distributed control [17]. Therefore, populating senescence effector pathways remains a major aim.

Kinome-focused siRNA screening has previously been used successfully to uncover pathways regulating cell immortality [18, 19]. In the current study, we undertook a large-scale siRNA screen combined with high-throughput imaging in the human melanoma cell line A375P to identify senescence effectors. Our results revealed diversity in levels of senescence engagement between gene targets and cell lines, consistent with recent data [4, 6]. Using a panel of HCT116 isogenic cell lines carrying common cancer-relevant gene mutations, we show that some senescence phenotypes can be engaged despite the absence of known effectors such as p53 or p21. However, distinct differences in proliferation arrest engagement are evident in different genetic backgrounds. Finally, we show senescence induction by knock-down of ECT2 in particular is greatly enhanced in the presence of oncogenic *KRAS*^*G13D*^. These findings suggest that pro-senescence therapy may be effective in various malignancies, including those harbouring oncogenic RAS mutations, which are common and often refractory to treatment. Furthermore, by populating the signalling pathways that regulate the senescent phenotype with the effectors identified by our screen, a more complete picture of the senescence response can be drawn.

## Results

### Identification of siRNAs that induce a senescent morphology in A375P melanoma cells

To identify potential senescence-inducing targets for drug discovery and extend our knowledge of senescence signalling in cancer, we initially performed validation of a high-content fluorescence imaging screen focused on senescence phenotypes. Using A375P cells treated with etoposide, we demonstrated that stable growth arrest in this cell line, assessed by colony formation assays and cell growth kinetics after compound washout, was accompanied by induction of a range of senescence markers. These included increased nuclear area, SAβGal and p21 expression, and 53BP1 and H2AX nuclear foci. We next tested an imaging assay based on the nuclear area marker and cell number in a small scale screen of 160 kinase inhibitor compounds by high content fluorescence imaging using the Operetta platform. We have previously reported the ability of some of these compounds to induce senescence in normal fibroblasts [20]. Four of the best hits from this screen were tested in a range of secondary assays, including colony formation assays, confirming the ability of screening based on these markers to identify chemical agents capable of causing a range of senescence phenotypes alongside stable proliferation arrest. These validation experiments are summarised in S2 File.

We next used the validated imaging assay to perform a large-scale screen of 10,414 gene targets using the Ambion Silencer Select Druggable Genome siRNA library. As quality controls on each screening plate, etoposide (S2 File) and an independent siRNA obtained from Qiagen against CDK1 (which is known to be involved in senescence) were included. Primary hits in A375P cells, detected by increased nuclear area and reduced proliferation compared to controls [20], were rescreened and ranked by confidence (Materials and Methods, S1 Fig) and a refined hit list of the 24 most robust hits and 16 others predicted to be druggable targets by functional analysis was generated for further validation. Criteria for the assessment of druggable targets included previous success in targeting the encoded protein or another of the same molecular class with a small molecule or drug-like compound, or the presence of a potentially druggable site within the protein structure. Many of the 24 highest-confidence hits were also classed as druggable, increasing our interest in them. The combined validation list of 40 targets showed a range of senescence-like morphologies upon knockdown, and included some weaker hits with fewer siRNAs passing the cut-off point (S1B Fig). These were included from a drug development standpoint and also to test a range of senescence responses in secondary validation assays. The siRNAs selected are given in Table 1, together with their prioritisation grouping and confidence ranking.

**Table 1:** siRNA selected for further investigation by priority group and confidence. Confidence ranking for nuclear area was based on number of standard deviations above the mean of plate negative controls and the number of library siRNA passing each threshold.

Target	Priority group	Confidence ranking
ACCN1	Druggable 16	1 siRNA at 3 x SD; 1 siRNA at 1 x SD
ALDOA	Top 24	2 siRNA at 3 x SD; 1 siRNA at 2 x SD
AURKB	Top 24	2 siRNA at 3 x SD
BUB1B	Top 24	3 siRNA at 3 x SD
CAPN11	Druggable 16	2 siRNA at 1 x SD
CAPN9	Druggable 16	2 siRNA at 1 x SD
CCNA2	Top 24	3 siRNA at 3 x SD
CDC45L	Top 24	2 siRNA at 3 x SD
CDC7	Top 24	2 siRNA at 3 x SD
CDK1	Positive control	
CHAF1B	Top 24	2 siRNA at 3 x SD; 1 siRNA at 2 x SD
CIT	Top 24	3 siRNA at 3 x SD
CRKRS	Druggable 16	1 siRNA at 3 x SD; 1 siRNA at 2 x SD
DDB1	Top 24	2 siRNA at 3 x SD
ECT2	Top 24	3 siRNA at 3 x SD
EFTUD2	Druggable 16	2 siRNA at 1 x SD
ESPL1	Top 24	3 siRNA at 3 x SD
GRIA3	Druggable 16	1 siRNA at 3 x SD; 1 siRNA at 2 x SD
INCENP	Top 24	3 siRNA at 3 x SD
KCNQ5	Druggable 16	1 siRNA at 3 x SD; 1 siRNA at 2 x SD
KIF11	Top 24	3 siRNA at 3 x SD
METAP2	Druggable 16	2 siRNA at 1 x SD
MMP24	Druggable 16	1 siRNA at 3 x SD
PABPN1	Top 24	2 siRNA at 3 x SD; 1 siRNA at 2 x SD
PDE3A	Druggable 16	1 siRNA at 3 x SD; 1 siRNA at 2 x SD
PSMA2	Top 24	2 siRNA at 3 x SD
PSMA5	Top 24	2 siRNA at 3 x SD; 1 siRNA at 2 x SD
PSMA7	Top 24	2 siRNA at 3 x SD; 1 siRNA at 2 x SD
PSMB1	Top 24	2 siRNA at 3 x SD
PSMB2	Top 24	2 siRNA at 3 x SD; 1 siRNA at 2 x SD
PSMB4	Top 24	2 siRNA at 3 x SD
PSMB5	Top 24	2 siRNA at 3 x SD; 1 siRNA at 2 x SD
RRM1	Druggable 16	3 siRNA at 1 x SD
RRM2	Druggable 16	2 siRNA at 1 x SD
SCN3B	Druggable 16	1 siRNA at 1 x SD
SMO	Druggable 16	2 siRNA at 1 x SD
TOP2A	Druggable 16	1 siRNA at 3 x SD; 1 siRNA at 1 x SD
TRRAP	Top 24	2 siRNA at 3 x SD; 1 siRNA at 2 x SD
UBL5	Druggable 16	1 siRNA at 3 x SD; 1 siRNA at 2 x SD
WEE1	Top 24	2 siRNA at 3 x SD
XPO1	Top 24	2 siRNA at 3 x SD; 1 siRNA at 2 x SD

**Fig 1:**
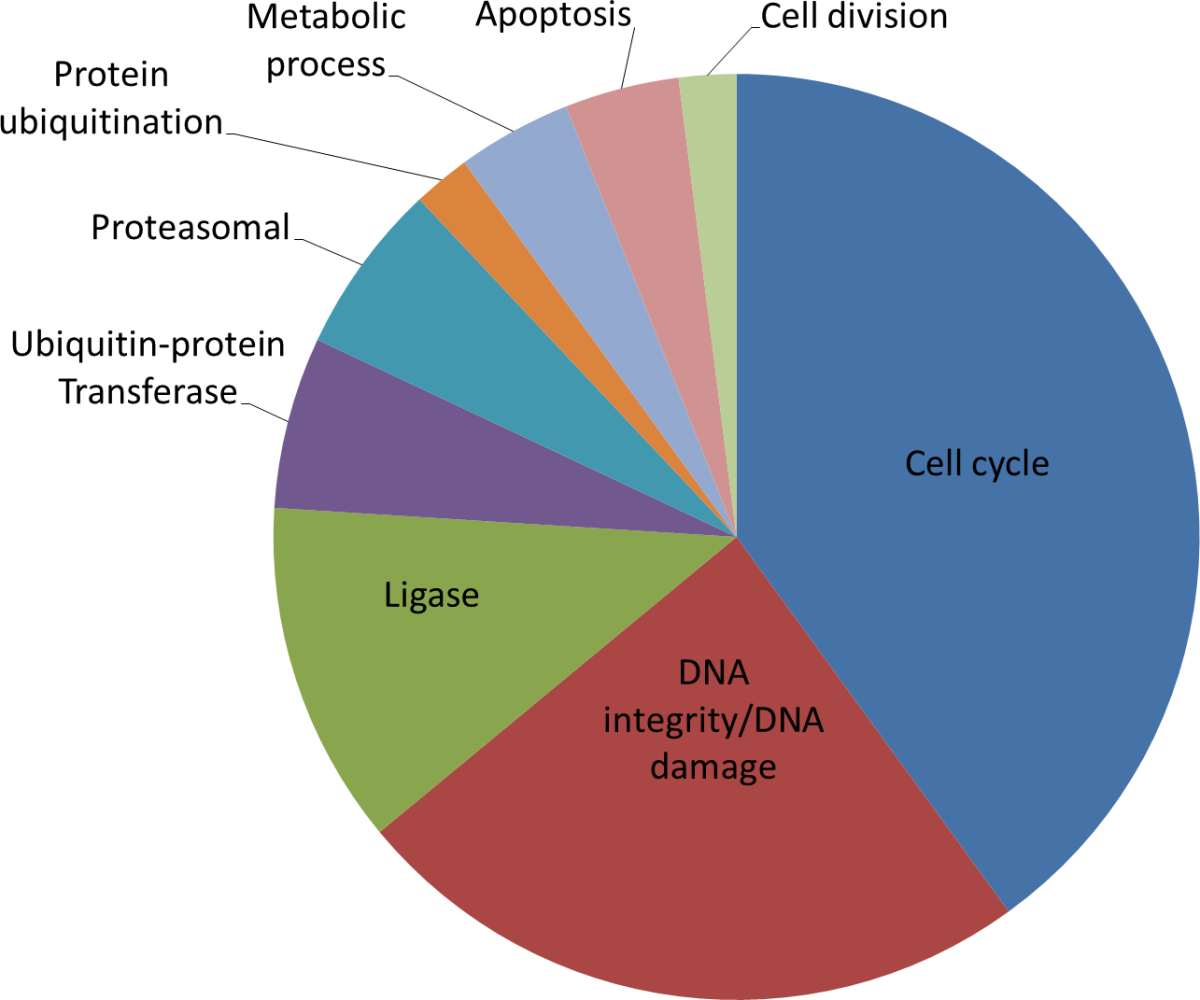
Gene ontologies associated with senescence inducing siRNA targets. An enrichment analysis for the gene ontology (GO) processes most significantly associated with the top 40 siRNA target genes was carried out in GeneGo from Metacore. The pie chart represents the number of GO processes falling into each of the 9 main categories expressed as a percentage of the 50 most significantly enriched GO processes.

Predictably, the majority of these 40 genes are involved in cell cycle regulation, the DNA damage response and maintenance of DNA integrity. However, several more interesting gene families emerged, including ribonucleoside-diphosphate reductase subunit genes RRM1 and RRM2, calpain cysteine protease family members CAPN11 and CAPN9 and several proteasome subunit family members (Fig 1). Interestingly, although the Qiagen positive control CDK1 siRNA performed as well as many of the top hits, the Ambion CDK1 siRNAs from the library were not in our top 40. We continued to use the Qiagen siRNA in further experiments.

### Screen hits induce expression of senescence biomarkers

**Fig 2:**
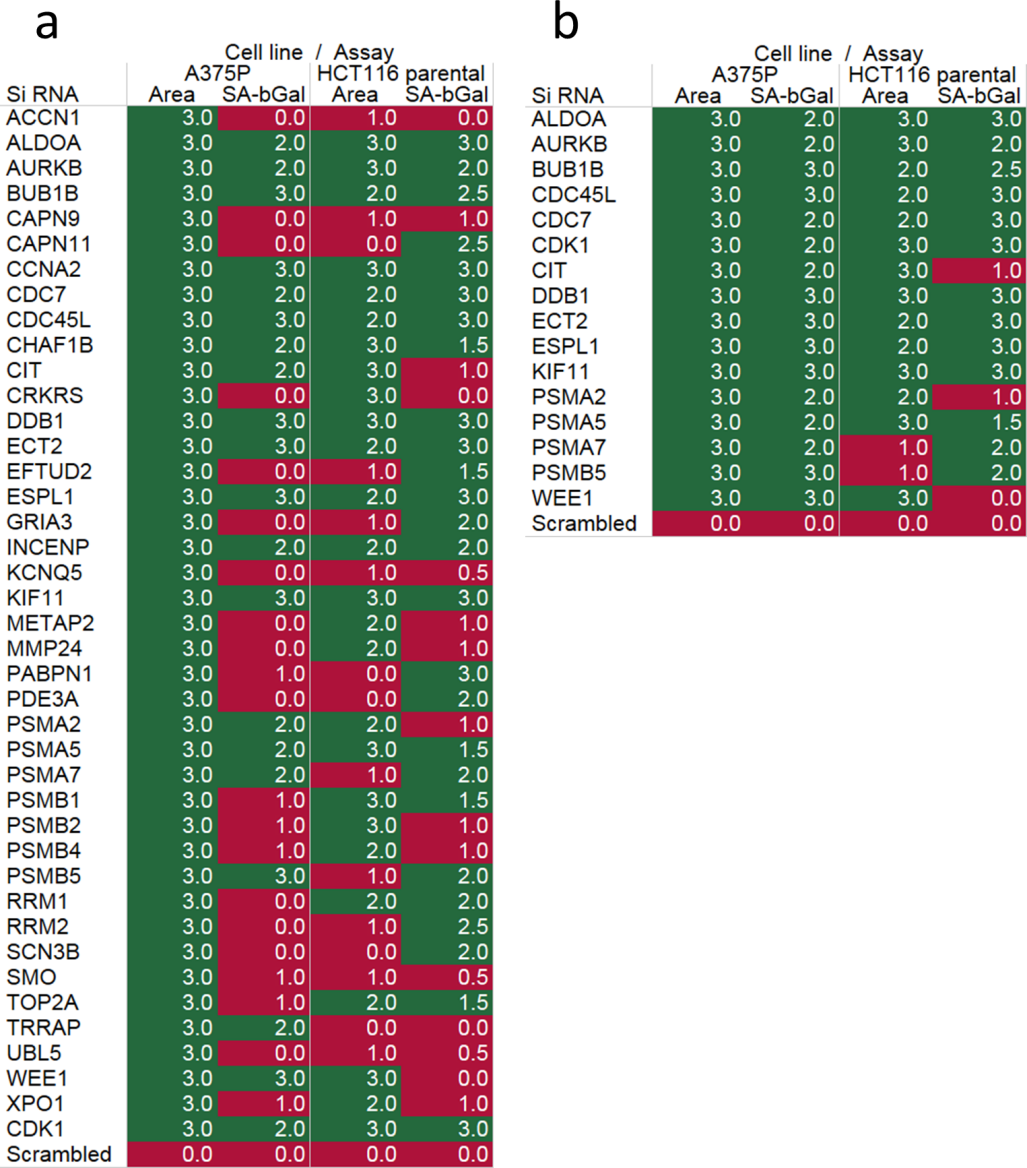
Expression of senescence biomarkers allows validation and refinement of screen hits. (a) Heatmap drawn in Tableau desktop representing hit (green) and miss (red) for mean nuclear area and SAβGal for the top 40 siRNA hits in A375P and HCT116. Hits were classed as ≥ 2/3 wells in ≥ 2/3 independent experiments passing the cut-off of mean scrambled control + 3 standard deviations. Values in heat-map boxes represent the number of repeat experiments in which at least 2/3 siRNA gave results greater than scrambled mean + 3SD. (b) Refined heatmap representing the 16 siRNA targets taken forward for further validation.

To further investigate the 40 prioritised siRNAs as senescence inducers, a fresh batch of the same siRNAs was tested for the ability to increase nuclear area and induce SAβGal in A375P cells using a stricter cut-off value of control mean + 3 standard deviations (SD) in at least 2/3 replicate wells in 2/3 independent experiments. Hereafter an “increase” will refer to an increase passing this criterion unless otherwise stated. All 40 siRNAs increased nuclear area in 3 independent experiments (Fig 2A & S2A Fig), further validating the primary screen results. However, many gave SAβGal levels below the stricter cut-off, suggesting a partial or atypical senescence response (Fig 2A & S2C Fig).

Analysis of these 40 siRNAs was extended to HCT116 colorectal carcinoma cells, chosen for the availability of isogenic derivatives with various oncogenic genotypes, previously used in screening [21] and their ability to express biomarkers of senescence (S2B and S2D Fig). The results with parental HCT116 cells further corroborated those for A375P (Fig 2A). Many of the strongest inducers of senescence in A375P also came out top in HCT116, while those that were negative for SAβGal in A375P were also negative for increased nuclear area and SAβGal in HCT116. Based on the SAβGal results for A375P, the nuclear area and SAβGal for HCT116, and the assessment of druggability, the list was refined to 16 siRNA targets for further analysis. This refined list encompassed many of the strongest hits from the siRNA screen. We continued to include the Qiagen positive control siRNA targeting CDK1 (Fig 2B).

**Fig 3:**
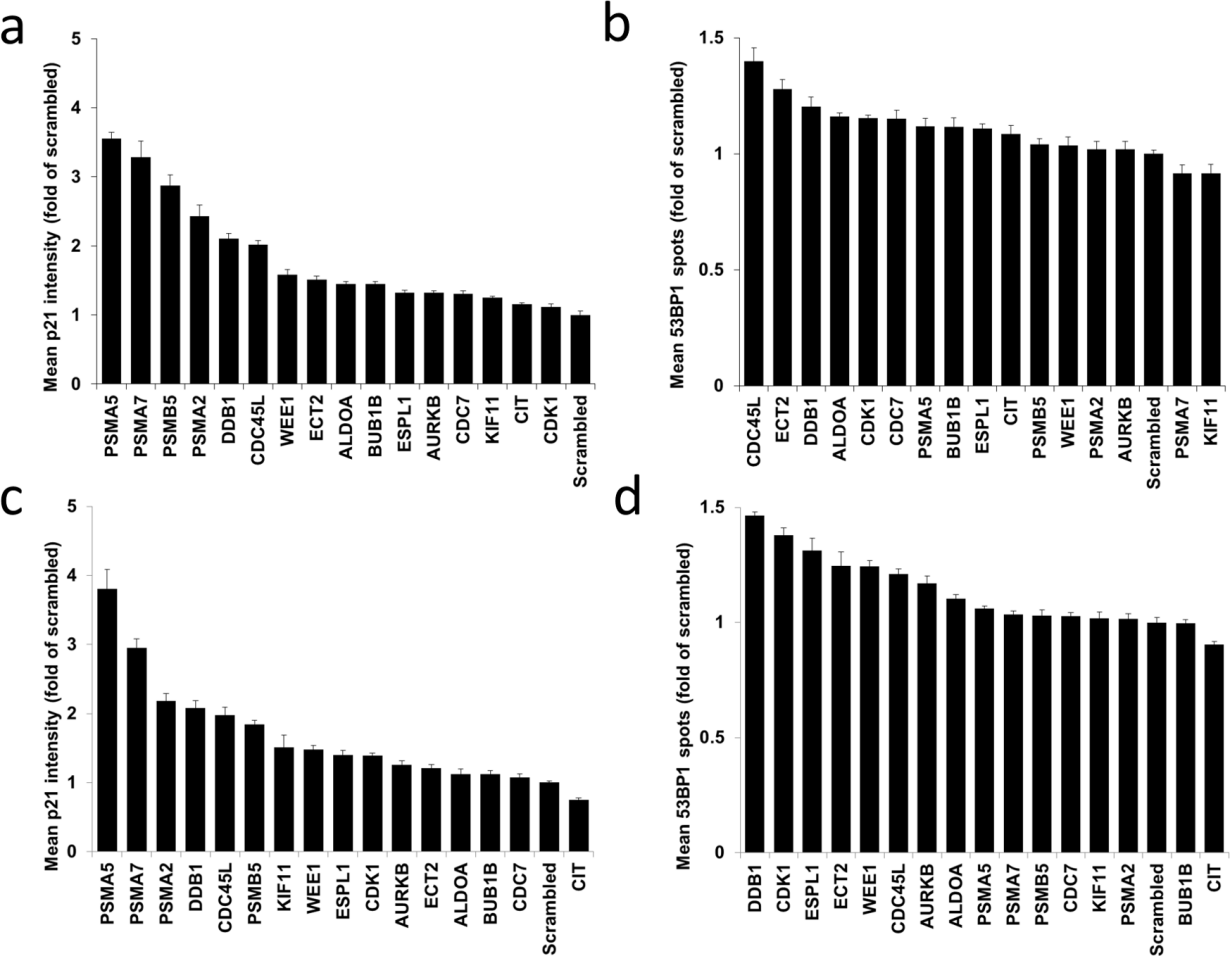
Expression of p21 and 53BP1 highlights a range of senescence responses to siRNAs. Expression of p21 induced by the top 16 siRNAs in A375P (a) and HCT116 (c) detected by immunofluorescent staining using the Operetta high content imaging platform. Expression was detected as mean nuclear intensity. Expression of 53BP1 foci induced by the top 16 siRNAs in A375P (b) and HCT116 (d) detected by immunofluorescent staining using the Operetta high content imaging platform. Expression was detected as mean spots (foci) per nucleus. Graphs represent the mean and standard error of triplicate wells from 3 independent experiments represented as a fold change of scrambled control.

Two additional biomarkers of senescence are p21 (CDKN1A) and recruitment of 53BP1 to sites of DNA damage (nuclear foci). To further characterise the response of the refined siRNAs we analysed induction of p21 and formation of 53BP1 foci by immunofluorescence in A375P and HCT116 cells transfected with the 16 hit and control siRNAs (Fig 3). Consistent with the results for nuclear area and SAβGal, all 16 test siRNAs induced expression of p21 greater than the non-targeting control in A375P (Fig 3A). In HCT116 cells, p21 expression was also increased by all test siRNAs except that targeting CIT (Fig 3C). Likewise, CIT siRNA failed to increase SAβGal in this cell line (Fig 2), and formation of 53BP1 foci (Fig 3D), suggesting that knockdown of CIT does not induce senescence in HCT116 cells. Generally, only a small induction of 53BP1 was evident in both cell lines (Fig 3B & 3D), however many of the top hits for nuclear area and SAβGal expression, including ECT2, ESPL1, DDB1 and CDC45L (Fig 2, S2 Fig & S3 Fig), were also the most positive for both p21 and 53BP1, suggesting a coordinated senescence response to the knockdown of these mRNAs.

### Hit siRNAs can induce senescence in cancer-specific mutant backgrounds

To investigate the ability of our top 16 siRNAs to engage senescence signalling in the presence of common oncogenic mutations, we used a panel of HCT116 isogenic cell lines (Table 2). The panel consisted of HCT116 parental (which has pre-existing *KRAS*^*G13D*^ and *PIK3CA*^*H1047R*^ heterozygous mutations), HCT116 *KRAS*^*+/-*^ (where the mutant *KRAS*^*G13D*^ allele has been knocked out), HCT116 *PIK3CA*^*+/-*^ (where the mutant *PIK3CA*^*H1047R*^ allele has been knocked out), HCT116 p21 null and HCT116 p53 null lines. Analysis of nuclear area and SAβGal expression in this panel showed mutation-specific variations in response. We also examined proliferation responses to these siRNAs. We defined cell number of 100%-150% of seeding density at day 5 post-transfection as cytostasis, greater than 150% as growth and less than 100% as toxicity.

**Table 2:** HCT116 isogenic cell panel.

Cell line	Genotype
HCT116 parental	*p53*^*+/+*^, *p21*^*+/+*^, *KRAS* ^*G13D/+*^, *PIK3CA*^*H1047R/+*^
HCT116 p21^-/-^	*p53*^*+/+*^, ***p21***^***-/-***^, *KRAS* ^*G13D/+*^, *PIK3CA*^*H1047R/+*^
HCT116 p53^-/-^	***p53***^***-/-***^, *p21*^*+/+*^, *KRAS* ^*G13D/+*^, *PIK3CA*^*H1047R/+*^
HCT116 KRAS ^+/-^	*p53*^*+/+*^, *p21*^*+/+*^, ***KRAS*** ^***+/-***^, *PIK3CA*^*H1047R/+*^
HCT116 PIK3CA ^+/-^	*p53*^*+/+*^, *p21*^*+/+*^, *KRAS* ^*G13D/+*^, ***PIK3CA***^***+/-***^

**Fig 4:**
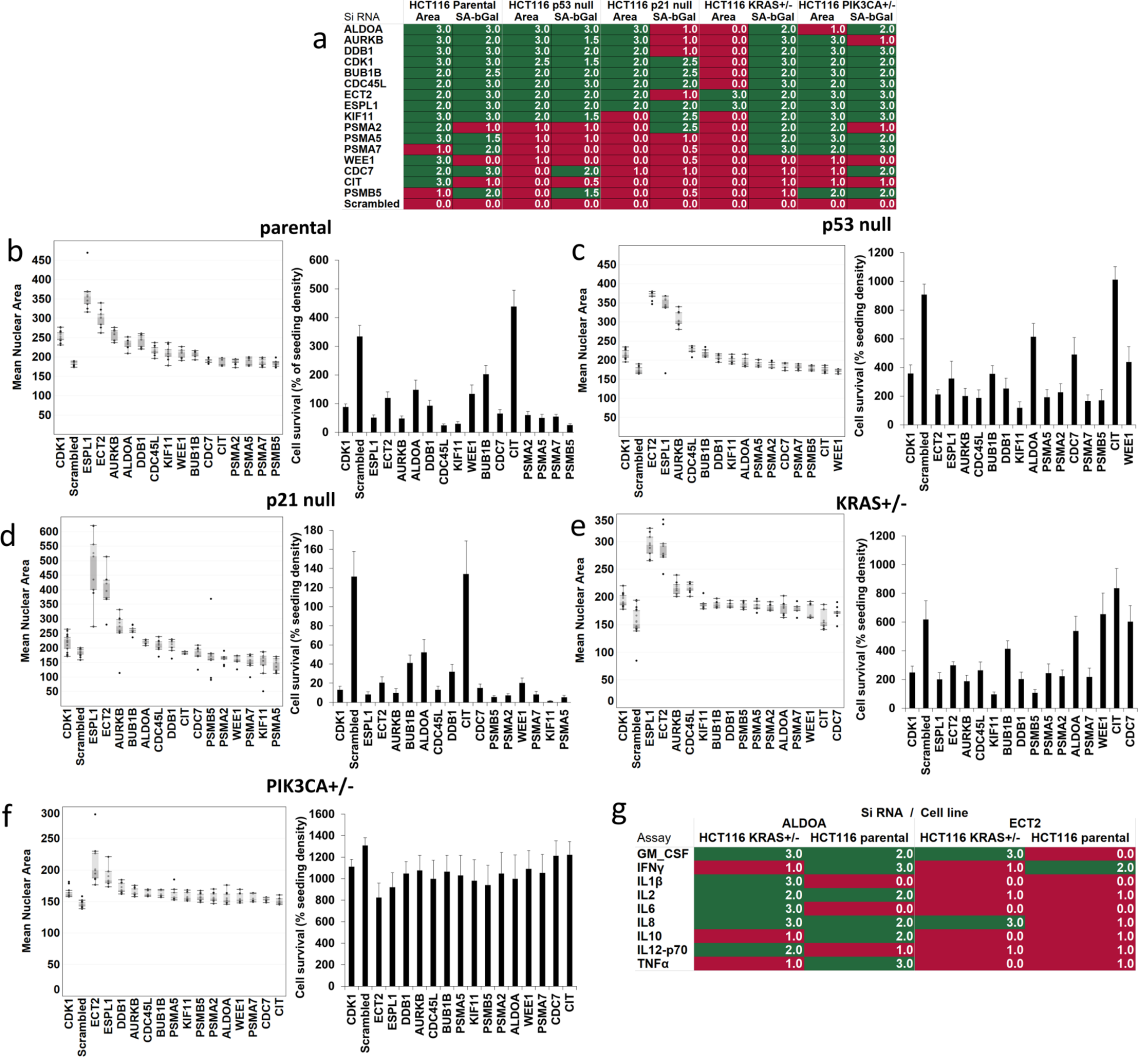
Senescence signalling and proliferation effects induced by siRNAs in the presence of common cancer-associated gene mutations. (a) Heat-map drawn in Tableau desktop representing hit (green) and miss (red) for mean nuclear area and SAβGal for the top 16 siRNA hits in HCT116 parental and isogenic derivatives. Hits were classed as ≥ 2/3 wells in ≥ 2/3 independent experiments passing the cut-off of mean scrambled control + 3 standard deviations. Values in heat-map boxes represent the number of repeat experiments in which at least 2/3 siRNA gave results greater than scrambled mean + 3SD. Mean nuclear area per well (μm^2^) in (b) HCT116 parental, (c) HCT116 p53 null, (d) HCT116 p21 null, (e) HCT116 KRAS^+/-^ and (f) HCT116 PIK3CA^+/-^ ranked in descending order for each cell line. Mean nuclear area per well (μm^2^) for triplicate wells in 3 independent transfections are represented as box whisker plots generated in Tableau desktop. Boxes represent the 25^th^– 75^th^ percentile of the data. Median level is shown as a colour change within the box. Positive (CDK1) and negative (Scrambled) siRNA controls are shown. Mean number of objects (nuclei) per well was used as a measure of proliferation, toxicity or cytostasis. Graphs represent the mean and standard error of mean number of objects from triplicate wells in 3 independent experiments expressed as a percentage of the initial seeding density (3000 cells). 100–150% was taken as no growth (cytostasis), <100% was taken as toxic. Inflammatory signalling was assessed using Mesoscale Discovery chemiluminescent ELISA (g). Hits were classed as ≥ 2/3 wells in ≥ 2/3 independent experiments passing the cut-off of mean scrambled control + 3 standard deviations. Values in heat-map boxes represent the number of repeat experiments in which at least 2/3 siRNA gave results greater than scrambled mean + 3SD.

We found 9/16 siRNAs in p53 null HCT116 cells and 4/16 siRNAs in p21 null cells that increased both nuclear area and SAβGal expression. A further 4 siRNAs increased nuclear area but not SAβGal expression in p21 null cells (Fig 4A, 4C & 4D). However, only 1 siRNA (KIF11) induced both phenotypes in addition to proliferation arrest as defined above in p53 null cells. A number of other siRNA did substantially reduce growth relative to scrambled control siRNA transfections, although the 100%-150% cutoff was not met (Fig 4C). In the HCT116 p21 null cells, transfection of the majority of the top 16 siRNAs, with the exception of CIT, resulted in cell numbers well below plating density (Fig 4D), suggesting that many cells died. However, the p21 null cells that remained viable after siRNA transfection expressed the other senescence phenotypes in many cases. It may be the case that this results from selective killing of non-senescent cells, while those that induce senescent phenotypes survive. However, further investigations would be required to determine this.

Conversely, the presence of *PIK3CA*^*H1047R*^ appeared to be required for a senescence response. Although 10/16 siRNAs induced both nuclear area increase and SAβGal expression in the *PIK3CA*^*+/-*^ cells (Fig 4A & 4F), we saw no reduction in proliferation with any of the siRNAs, indicating that true senescence was not induced in response to knock-down of these targets in *PIK3CA*^*+/-*^ cells (Fig 4F). These results are summarised in Table 3, where we classify each siRNA as provoking a senescence response (increased nuclear area, increased SaβGal and proliferation arrest) or partial responses, involving some combination but not all of these phenotypes. Overall, most responses were only partial, suggesting that a spectrum of “senescence-like” phenotypes can be induced in cancer cells of different backgrounds.

**Table 3:** Genotype-specific senescence responses in HCT116 isogenic cell lines.

Cell genotype	Senescence response	Partial senescence response
Parental	ALDOA, ECT2	AURKB, DDB1, CDK1, BUB1B, CDC45L, ESPL1, KIF11, PSMA2, PSMA5, PSMA7, WEE1, CDC7, CIT, PSMB5
p21-/-	None	CDK1, BUB1B, CDC45L, ESPL1, ALDOA, AURKB, DDB1, ECT2, KIF11, PSMA2
P53-/-	KIF11	ALDOA, AURKB, DDB1, CDK1, BUB1B, CDC45L, ECT2, ESPL1, CDC7, PSMB5
KRAS+/-	None	ECT2, ESPL1, ALDOA, AURKB, DDB1, CDK1, BUB1B, CDC45L, KIF11, PSMA2, PSMA5, PSMA7
PIK3CA+/-	None	DDB1, CDK1, BUB1B, CDC45L, ECT2, ESPL1, KIF11, PSMA2, PSMA5, PSMA7, PSMB5, ALDOA, AURKB, PSMA2, CDC7

Parental HCT116 cells showed “complete” senescence response to 2 siRNAs: ECT2 and ALDOA. Interestingly, the siRNA targeting ECT2 induced among the strongest increases in nuclear area across all of the isogenic HCT116 lines tested, along with siRNA against ESPL1 (Fig 4B–4F). Knockdown of ECT2/ESPL1 also resulted in increased expression of SAβGal in all cell lines except p21 null cells (Fig 4A). Furthermore, these were the only siRNAs to increase nuclear area and SAβGal expression in the HCT116 *KRAS*^*+/-*^ line (Fig 4A & 4E), in contrast to parental and other HCT116 cells, which showed partial senescence responses involving these phenotypes to many of the siRNAs. Comparing responses, a more robust phenotype also including cytostasis was observed for both ECT2 and ALDOA in the presence of mutant *KRAS* (parental cells), whereas only partial responses were observed in *KRAS*^*+/-*^ cells (Table 3). For ECT2, both nuclear area and SAβGal expression were induced in *KRAS*^*+/-*^ cells, but proliferation arrest did not occur. For ALDOA, only SaβGal induction was observed in *KRAS*^*+/-*^ cells.

To further investigate these potential *KRAS* dependent effects on the senescence response, we characterised the SASP of parental and *KRAS*^*+/-*^ cells after transfection with these siRNAs (Fig 4G). Mutliplex chemiluminescent ELISA assays were performed to detect nine pro-inflammatory cytokines. The same cut-off of greater than mean + 3SD of scrambled control by at least 2/3 siRNA in 2/3 experiments was used for assignment of increase. ALDOA knockdown caused a complex inflammatory phenotype involving increased levels of GM-CSF, IL2 and IL8 in both cell lines. In the absence of mutant *KRAS*, IL1β, IL6, and IL12-p70 were also induced. In parental cells, instead, IFNγ and IL10 were upregulated. In contrast, ECT2 knockdown produced less complex effects. In *KRAS*^*+/-*^ cells, GM-CSF and IL8 were induced. In the presence of mutant *KRAS*, only IFNγ was increased.

We additionally performed assays for activated caspase 3/7 in parental HCT116 cells after ECT2 knockdown (S4A Fig). While the positive control CDK1 siRNA increased levels by 1.8-fold, ECT2 knockdown did not increase activated caspase 3/7. Examination of the levels of ECT2 mRNA detected in microarray analysis after transfection confirmed that the message was reduced to 45% of the level of scrambled control in parental cells (S4B Fig). Taken together, these results indicate that ECT2 induces multiple markers of senescence including growth arrest without caspase activation specifically in *KRAS* mutant cells, while inducing a SASP phenotype that may suggest sensitisation to apoptotic triggers due to the known involvement of IFNγ in death receptor signalling [22].

### Knock-down of ECT2 can induce senescence markers in a *KRAS*^*G13D*^ dependent manner

ECT2 is a guanine nucleotide exchange factor (GEF) for the RHO family of GTPases and has been associated with regulation of RAS–MAPK signalling [23]. This and the induction of a more robust senescence phenotype in the presence of mutant *KRAS*^*G13D*^ led us to focus on ECT2. To further investigate the relationship between the senescence response to ECT2 down-regulation and the *KRAS*^*G13D*^ mutation, we tested the spontaneously immortalised non-transformed mammary epithelial cell line MCF10a and its isogenic derivative MCF10a *KRAS*^*G13D/+*^, in which the *KRAS*^*G13D*^ allele is knocked-in at the endogenous locus. We also analysed an additional colorectal cancer cell line DLD1, which, like HCT116, has a *KRAS*^*G13D*^ mutation, and an isogenic counterpart in which the mutated *KRAS* allele is knocked out to leave only wild-type KRAS (DLD1 *KRAS*^*+/-*^).

**Fig 5:**
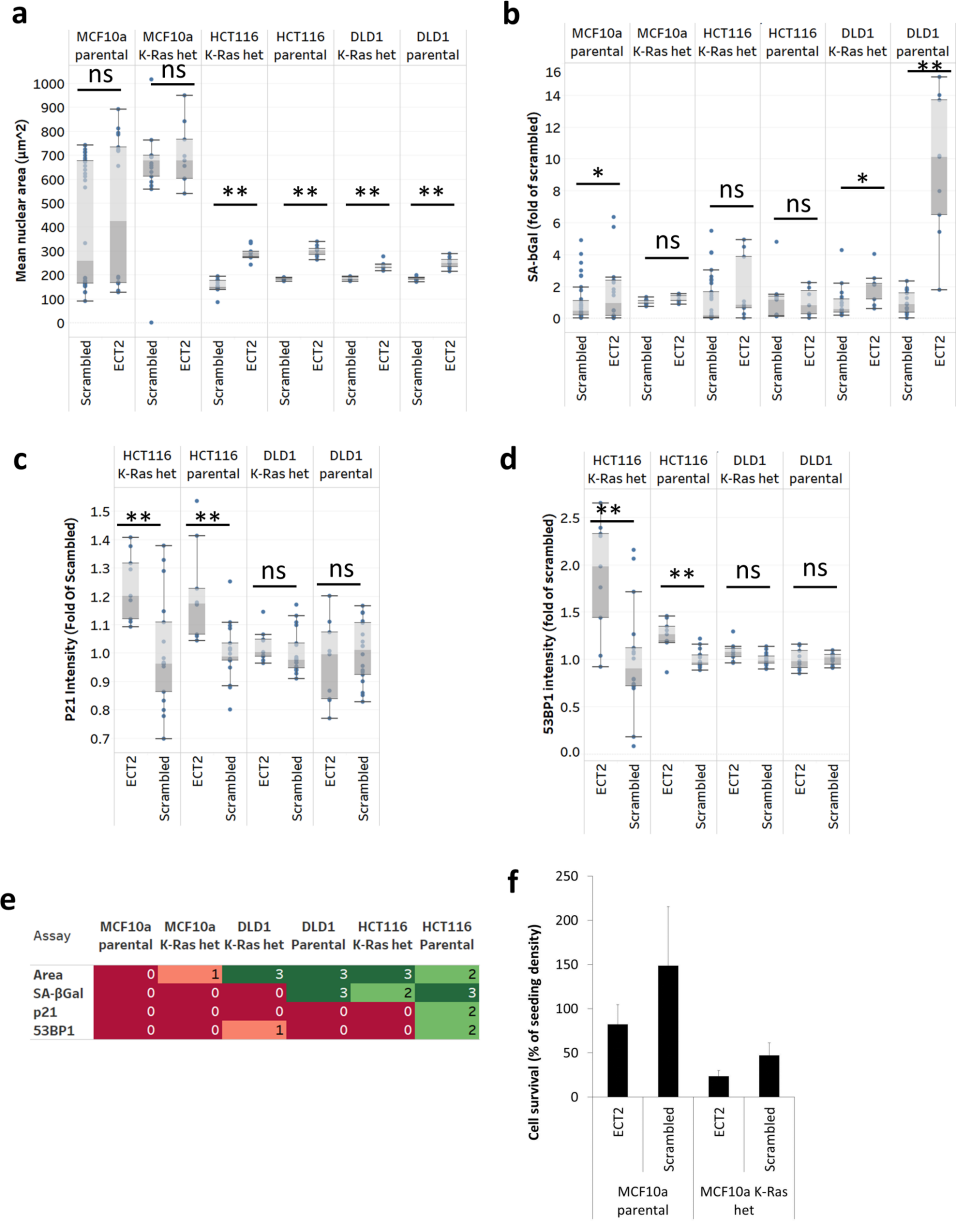
Senescence engagement is more robust in the presence of activating *KRAS*^*G13D*^ mutation in colorectal carcinoma cell lines. (a) Mean nuclear area per well (μm^2^), (b) mean SAβGal expressed as fold of scrambled for each individual cell line, (c) mean p21 nuclear intensity expressed as fold of scrambled for each individual cell line, (d) mean 53BP1 spots (foci) per nuclei expressed as fold of scrambled for each individual cell line. Box whisker plots generated in Tableau desktop represent triplicate wells in 3 independent transfections. Boxes represent the 25^th^– 75^th^ percentile of the data. Median level is shown as a colour change within the box. P-values were calculated by 2-tailed Student’s T-test in Excel assuming unequal variance: (*) p<0.05, (**) p<0.001. (e) heat-maps drawn in Tableau desktop representing hit (green) and miss (red) for mean nuclear area, SAβGal, p21 and 53BP1 in MCF10a parental and *KRAS*^*G13D/+*^ knock-in cells compared to HCT116 and DLD1 parental and *KRAS*^*+/-*^ G13D knock-out cells transfected with siRNA targeting ECT2. Hits were classed as ≥ 2/3 wells in ≥ 2/3 independent experiments passing the cut-off of mean scrambled control + 3 standard deviations. Values in heat-map boxes represent the number of repeat experiments in which at least 2/3 siRNA gave results greater than scrambled mean + 3SD. Mean number of objects (nuclei) per well was used as a measure of proliferation, toxicity or cytostasis (f). Graphs represent the mean and standard error of mean number of objects from triplicate wells in 3 independent experiments expressed as a percentage of the initial seeding density (3000 cells). 100–150% was taken as no growth (cytostasis), <100% was taken as toxic.

Combined results from all repeats of each individual ECT2 siRNA transfection in this focussed cell panel showed significant increases in nuclear area in all cell lines except the MCF10a isogenic pair (Fig 5A). We did see a slight but significant increase in SAβGal expression in parental MCF10a cells with ECT2 knock-down (Fig 5B). SAβGal expression was significantly increased in response to ECT2 knock-down in the presence and absence of *KRAS*^*G13D*^ in DLD1 cell lines, but the increase did not reach significance in the HCT116 lines; this effect was strongest in the DLD1 parental cells with the *KRAS*^*G13D*^ mutation (Fig 5B). Expression of p21 (Fig 5C) and 53BP1 (Fig 5D) was also significantly increased upon ECT2 knockdown in the HCT116 cell lines, confirming a senescence response.

**Fig 6:**
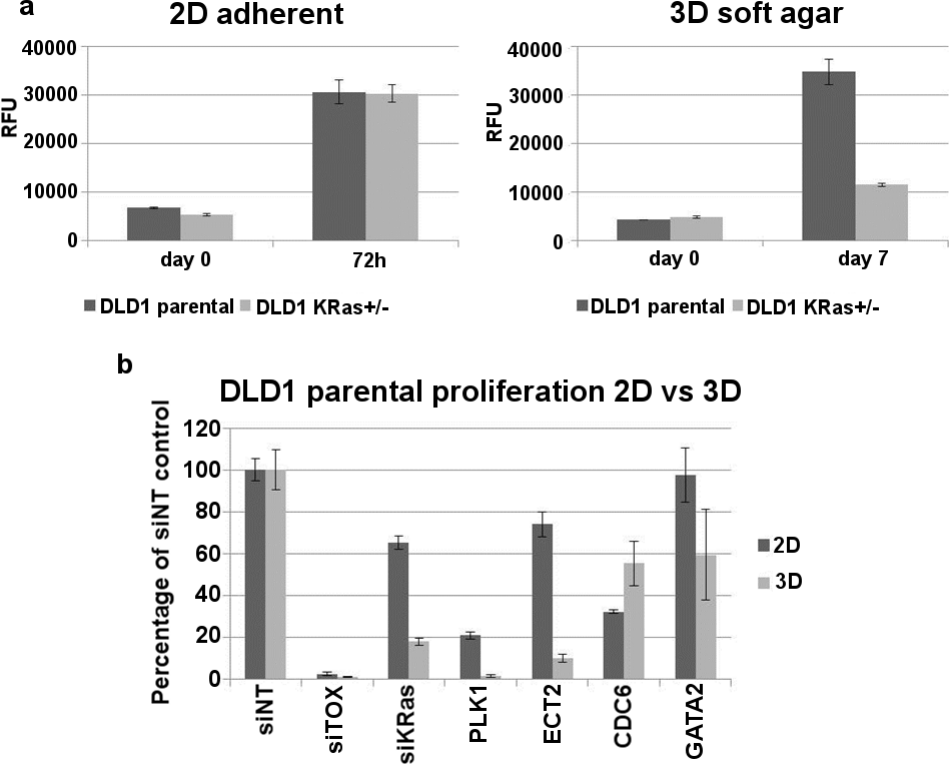
ECT2 targeting siRNAs inhibit the growth of DLD1 parental cells in 2D and 3D culture conditions. (a) DLD1 parental and *KRAS*^*+/-*^ cells were cultured for 72h (2D) or 7 days (3D) before proliferation was quantified using Alamar Blue (10% v/v). DLD1 *KRAS*^*+/-*^ cells in which the *KRAS*^*G13D*^ allele had been knocked out proliferated poorly on soft agar. Bars represent the mean and standard error of 3 technical replicates. (b) DLD1 parental cells were screened in 2D (72h) and 3D (7d) following transfection with siRNAs and proliferation was assessed using Alamar Blue (10%v/v). Bars, expressed as a percentage of the proliferation detected in cells transfected with the non-targeting control siRNA, represent the mean and standard error of 6 technical replicates.

Analysing the results according to the cut-off values used previously (at least 2/3 siRNAs increase marker levels beyond mean of scrambled + 3SD in at least 2/3 experiments), we used heat-maps to summarise the expression of all 4 senescence biomarkers in our *KRAS*^*G13D*^ isogenic cell line panel. In the non-transformed MCF10a isogenic pair ECT2 knock-down did not reproducibly induce the expression of any biomarker, whereas in HCT116 cell lines the increase in nuclear area and expression of SAβGal was detected in at least 2 out of 3 experiments in both the parental and the *KRAS*^*+/-*^ cells, as before. High levels of p21 and 53BP1 were detected in HCT116 parental cells, but no positive increase in *KRAS*^*+/-*^ cells, suggesting a more robust senescence response in the presence of oncogenic *KRAS*^*G13D*^. This was further corroborated in the DLD1 cells (Fig 5E), although no significant induction of p21 or 53BP1 was detected in these cells. Analysis of MCF10a proliferation showed a low level of toxicity as defined previously (83% survival), while scrambled transfectants grew to only 149% of seeding density. As noted, this mild toxicity occurred without evidence of induction of any senescence markers. *KRAS* mutant cells were sensitive to transfection (47% survival after transfection with scrambled siRNA). However, the relative reduction with ECT2 was similar (24% of seeding density), again without induction of senescence markers (Fig 5F). Therefore, in this cell panel, engagement of senescence in *KRAS* mutant background also appeared to be specific for cancer cells. Further studies will be required to evaluate the relative sensitivity of a wider range of normal and *KRAS* mutant cancer cells. It is known that DLD1 are particularly reliant on mutant KRAS signalling for proliferation in soft agar (surrogate 3D conditions) compared with standard tissue culture 2D conditions. HCT116 cells display a similar phenotype, although the difference between WT/mut (parental) and WT/- (KRAS +/-) genotypes is far less pronounced than DLD1 [24]. We therefore analysed the more sensitive DLD1 system to determine whether ECT2 knockdown affects this phenotype. In our hands, DLD1 *KRAS*^*+/-*^ cells proliferated poorly in soft agar, although they grew normally on tissue culture plastic as expected (Fig 6A). We examined the link between KRAS and ECT2 in these conditions. Knockdown of KRAS reduced proliferation in DLD1 parental cells grown in 2D and 3D, but had a greater effect in 3D conditions, as did other published KRAS synthetic lethal targets such as PLK1 and GATA2 (Fig 6B) [25, 26]. ECT2 knock-down in DLD1 parental cells reduced proliferation modestly in 2D, but substantially in 3D conditions, to around 10% of the non-targeting control (Fig 6B), consistent with a senescence response in these conditions, which better model growth in vivo.

**Fig 7:**
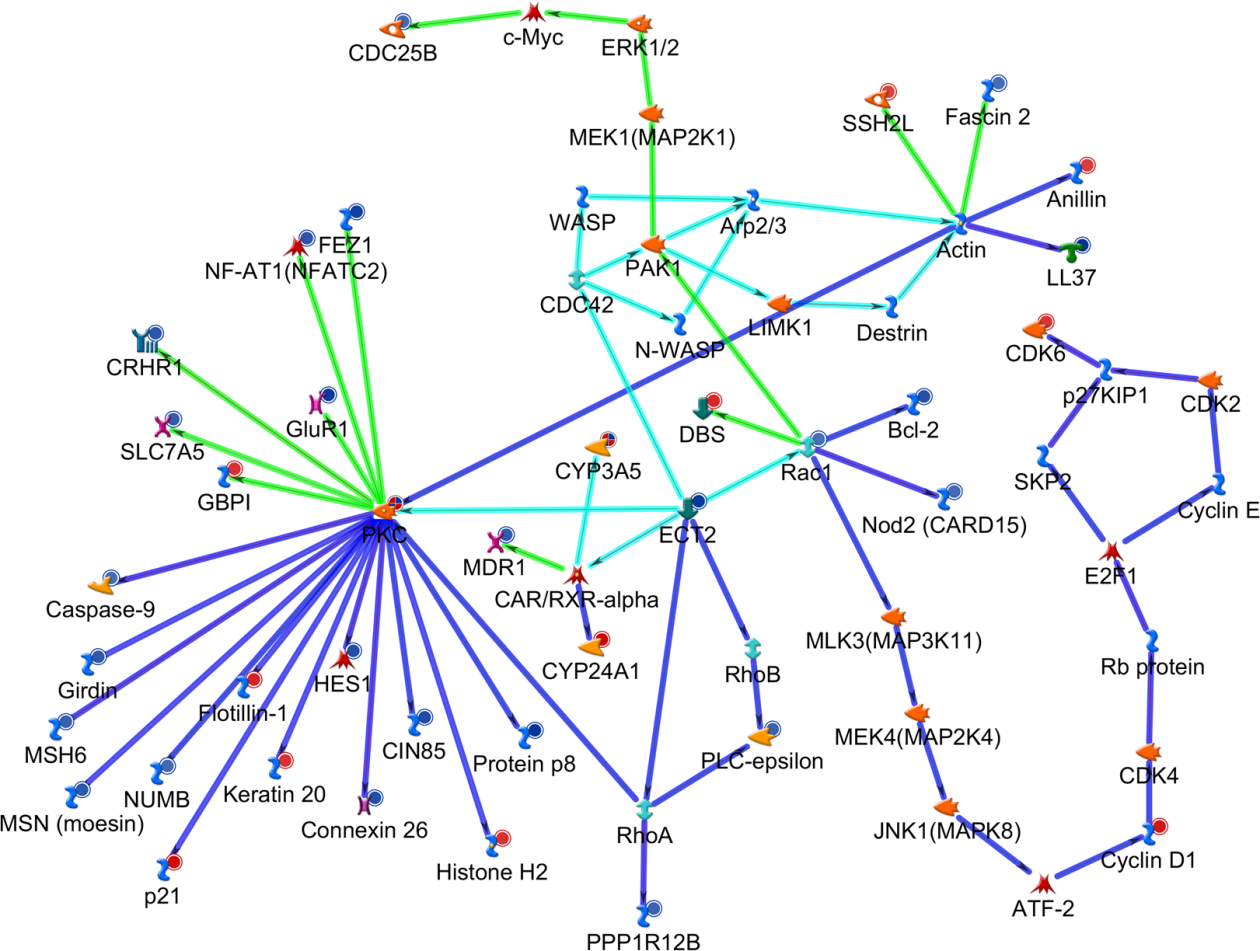
Network analysis suggests a more robust senescence phenotype is induced in the presence of activating *KRAS*^*G13D*^ mutation. Signalling network built out from ECT2 as a seed object using the Shortest Paths algorithm in GeneGo from Metacore. Gene expression data from HCT116 parental and HCT116 KRAS^+/-^ cells was overlaid on this network. Green lines represent HCT116 parental-specific paths, dark blue represent HCT116 KRAS^+/-^ specific paths, light blue represent common paths. Red circles next to icons reflect up-regulation, while blue circles reflect down-regulation in expression profiles. Shading intensity indicates fold change (minimum 2-fold). Icons indicate protein functional classes.

Finally, to investigate the signalling networks underlying the senescence response to ECT2 knock-down in the presence or absence of *KRAS*^*G13D*^, we used network modelling to interrogate gene expression profiles of HCT116 parental and *KRAS*^*+/-*^ cells transfected with siRNA targeting ECT2. A directed network built out from ECT2 using the Shortest Paths algorithm in MetaCore from GeneGo showed shared pathways associated with cell cycle regulation and cytoskeletal remodelling, diverging between the two lines at key hubs that included actin, PKC and RAC1, a RAS superfamily GTPase (Fig 7). Diverging networks show reduced signalling from RAC1 towards MYC in HCT116 parental cells, leading to down-regulation of CDC25B and cell cycle arrest, consistent with the robust senescence response. In HCT116 *KRAS*^*+/-*^ cells, signalling from RAC1 leads to an up-regulation of cell cycle signalling through cyclin D1 to CDK6. Expression of CDK6 is associated with G1/S progression; this might contribute to a weaker senescence response, although this warrants further investigation.

## Discussion

The concept of inducing senescence for cancer treatment is an attractive one that has gained interest recently. The presence of residual senescence signalling in various advanced tumour types [4] and the detection of senescence biomarkers in response to standard chemo- and radiotherapy regimens [2] suggests that pro-senescence therapy, either alone or in combination with established therapies, may improve outcomes [3]. Furthermore, the presence of senescence biomarkers in pre-malignant or benign lesions suggests that novel agents that are able to induce senescence in tumour cells might have reduced systemic toxicity and fewer side-effects than standard treatments, improving prognosis where senescence can be robustly achieved.

To identify and realise the potential of pro-senescence therapies we need to understand the complex nature of senescence signalling, especially in cancer cells, and to identify robust biomarkers of this response. Here we describe the identification and validation of a panel of siRNAs that induce a spectrum of senescence responses in two cancer cell lines, A375P melanoma and HCT116 colorectal carcinoma. Many of the top siRNA hits targeted genes were involved in cell cycle regulation and maintenance of DNA integrity, consistent with the senescence phenotype. Proteasomal and ubiquitin-protein transferase activities also featured strongly, with a number of proteasome family members in the siRNA target list. This is consistent with reduced proteolytic activity and down-regulation of proteasome β-catalytic subunits reported in human primary fibroblasts senescing *in vitro* and in ageing human tissues [27]. Moreover, the treatment of cultured primary fibroblasts with specific proteasome inhibitors induces a senescence-like phenotype that includes irreversible growth arrest and expression of SAβGal [28]. In addition we found that siRNA knock-down of AURKB induced nuclear size increase and expression of SAβGal in both A375P and HCT116 cells, consistent with and confirming the results of a parallel compound-based screen for senescence effectors in IMR90 human diploid fibroblasts [20]. Furthermore, siRNA knock-down of AURKB, BUB1B, ECT2, INCENP, KIF11 and CIT induced large nuclear morphologies that were associated with mitotic defects and growth arrest in a large-scale phenotypic screen in HeLa cells [29].

To validate the observed responses, we investigated the expression of two other established senescence biomarkers, senescence effector p21 [8], and DDR factor 53BP1, in 16 of the most robust siRNA hits in both A375P and HCT116 cells. DNA damage components are widely used as senescence markers [6, 30]. The extent of senescence response following gene knockdown depended on both the genetic background and cell type. Some siRNAs, including those targeting DDB1, PSMA5 and ECT2, induced a robust senescence response with expression of all 4 biomarkers in both cell types, while others, such as those targeting CIT, induced a weak response involving only increased nuclear area and SAβGal expression in A375P and little to no induction of p21 or 53BP1 in either cell line. This situation is consistent with a partial senescence response where genes might contribute to senescence evasion but are not crucial [31].

Indeed, considering effects on cell proliferation, a large percentage of hits could induce combinations of senescence associated phenotypes in cell lines lacking key normal senescence effectors p53 (commonly mutated in various cancer types) or p21. Senescence in a p21 null background has previously been shown in murine fibroblasts [32, 33]; and in HCT116 isogenic cell lines the plant tannin gallotannin was shown to induce a senescence response independent of p53 or p21 expression [34]. Similarly neonatal human melanocytes senesce without expressing p53 or p21, just p16 [35] and benign nevi likewise have p16 and rarely express p21 [36]. However, in our experiments, induction of the full spectrum of markers including growth arrest was limited to KIF11 in the p53 deficient background, while effects on cell number consistent with toxicity were observed in most cases in the p21 null background. It is well known that many existing toxic and targeted agents are capable of causing senescence in a subset of cells, despite the main phenotype caused being cell death, and this may be the most common mode of senescence induction in p21 null cells. The senescent phenotype results from the complex co-operative interaction of various signalling pathways, so it is not surprising that, in the right context, cells with a particular defect can still respond at least partially [37]. Our study therefore highlights the spectrum of “senescence-like” phenotypes that can arise in different mutational contexts.

Activating mutations in *KRAS* have been described in various cancer types including non-small cell lung and pancreatic cancers and in around 50% of colorectal carcinomas [38]. However RAS proteins have appeared undruggable until recently [39]. Thus research has focussed on targeting RAS signalling pathways and downstream effectors [38] including *BRAF*^*V600E*^ and PI3Kα (PIK3CA). Ten of our 16 top siRNA hits in HCT116 cells in which *PIK3CA*^*H1047R*^ was deleted induced senescence markers, yet none of them arrested proliferation, notably suggesting dependence of the actual arrest upon oncogenic *PIK3CA* in HCT116 cells. Analysis of the response in the HCT116 isogenic line lacking the activating *KRAS*^*G13D*^ mutation revealed that only siRNAs targeting ALDOA and ECT2 were capable of inducing SAβGal and nuclear area in this background. ECT2 also reduced proliferation in this background, though not as robustly as the parental cells.

ECT2 (epithelial cell transforming 2) is a known oncogene that is upregulated in various cancer types [40] and associated with poor prognosis in glioblastoma [41] and astrocytoma [42]. Prognostic significance of elevated ECT2 mRNA expression has been evaluated and positively correlated with protein expression in a range of cancer types [43, 44]. Its regulation is relatively understudied. However, ECT2 expression is known to be transcriptionally regulated through the cell cycle and during DNA damage to control mitosis [45, 46]. Regulation of ECT mRNA stability via FXR1 may also contribute to FXR1 oncogenic effects [47], while the tumour suppressor effects of miR-223 may be partly mediated by targeting the ECT2 3’UTR [48].

From a therapeutic standpoint, siRNA knock-down of ECT2 in glioblastoma cell lines caused decreased proliferation, migration and invasion [49], and mice with U251 astrocytoma cell line xenografts expressing ECT2 shRNAs showed significantly greater survival than non-targeting shRNA controls [42], making it an interesting target. Our druggability assessment for ECT2 showed no known drugs available to target this gene or other family members; however the protein structure is available and revealed potential druggable sites that could be exploited for drug development. It will be of interest in future studies to develop inducible ECT2 knockout systems to more thoroughly investigate the mechanisms and downstream effects of senescence induction when all cells in the population are targeted.

ECT2 regulates cell fate in *C*. *elegans* through RAS–MAPK signalling via crosstalk from the RHO-1 pathway [23]. Here, knockdown of ECT2 induced a more complete senescence response in HCT116 cells with *KRAS*^*G13D*^, but not in MCF10a non-neoplastic breast epithelial cells irrespective of *KRAS*^*G13D*^, suggesting a possible tumour-specific effect. Furthermore, SaβGal was significantly induced in DLD1 parental line with *KRAS*^*G13D*^ in comparison to the KRAS^+/-^ derivative. These links between ECT2 and RAS signalling make this an attractive and novel target worthy of further investigation for pro-senescence therapy of *KRAS* mutant tumours. Moreover ECT2 was recently identified in a large siRNA screen as being required for the survival and proliferation of KRAS transformed HCT116 cells, but not those lacking mutant *KRAS* [25]; and knockdown of KRAS, ECT2 and other KRAS synthetic lethal targets PLK1 and GATA2, in parental DLD1 cells substantially reduced growth in 3D in our hands.

We used network analysis to show that knockdown of ECT2 leads to further mRNA changes in a large number of genes closely associated with its signalling pathway. A similar approach was taken by Long and colleagues to infer ECT2 signalling mechanisms in pancreatic cancer [50]. Our findings point to significant downregulation of Rac1 and PKC signalling, both of which are in line with the known signalling functions of ECT2, in addition to upregulation of p21 and modulation of multiple other cell cycle and cytoskeletal genes such as vimentin and cdc25. Together, these results are in line with the effects on senescence that we observed in our screening assays.

The screen described here provides proof-of-concept of the ability to induce and detect novel senescence effectors in cancer cells, some with actions enhanced by common oncogenic mutations. Consistent with recent observations, a range of senescence responses was detected, highlighting the importance of using multiple phenotypes to measure the extent of the response induced [3, 6]. One of our most robust hits, ECT2 siRNA, had the ability to engage a senescence response enhanced by mutant *KRAS*^*G13D*^ and therefore shows promise as a target for senescence induction therapy for a range of cancers harbouring this mutation. However, to realise the full potential of candidate targets and markers identified in screening, appropriate routes to translation need to be established. We have previously investigated plasma markers of human ageing and DNA damage in gastrointestinal adenocarcinoma patients [51]. Furthermore, we have recently initiated two large, prospective, longitudinal studies to evaluate multiple candidate senescence biomarkers including ECT2 (UK Clinical Research Network trials 12434 and 12435). Ultimately, validation of novel markers and targets in well-defined human cancer populations will be required to accelerate the field of senescence therapeutics.

## Materials and methods

### Image analysis

Transfections were performed as described in S1 File. 5 days after transfection cells were fixed in 4% paraformaldehyde (PFA) or 0.5% glutaraldehyde (pH 7.2) and stained for analysis of nuclear area, p21, 53BP1 or SAβGal respectively. Images were captured on the Operetta high content imaging system (PerkinElmer) at 10x magnification and 9 fields of view per well and data analysed using Harmony software (PerkinElmer) and the parameters outlined in table A of S1 File for nuclear area, p21 and 53BP1. For all analyses nuclei were detected using a modified “find nuclei” algorithm using method B (individual threshold 0.40, common threshold 0.40, contrast >0.10) and split factor 4.4. Border objects were excluded to ensure only whole nuclei were analysed and size criteria to detect DAPI stained objects >40μm^2^ and <4000 μm^2^ were applied. Images of SAβGal stained cells were captured on the ArrayScan (Thermo Fisher Scientific) using the parameters outlined in table B of S1 File.

### Immunofluorescence

Transfections were performed as described in S1 File. 5 days after transfection cells were fixed with 4% PFA, permeabilised in 0.2% Triton x-100 in PBS and blocked in 10% normal goat serum with 1% BSA. Cells were dual immunostained with primary antibodies against p21 and 53BP1 (1:100 in 1% BSA in PBS) and incubated on a rocking platform at 4°C overnight. Alexa Fluor secondary antibodies were combined at 1:200 dilution in 1% BSA in PBS and incubated on the cells for 1hr at room temperature protected from light. Cells were counterstained with 0.1μg/ml DAPI dilactate for 5 min at room temperature and stored at 4°C protected from light. Images were captured on the Operetta high content imaging system (PerkinElmer) at 10x magnification and 9 fields of view per well and data analysed using Harmony software (PerkinElmer) using the parameters outlined in table A of S1 File. For all assays DAPI stained nuclei were defined as described for nuclear area and this population was then selected for analysis of p21 and 53BP1 as described in table A of S1 File. For MCF10a parental and KRAS^G13D^ lines images were captured on the ArrayScan (Thermo Fisher Scientific) at 20x magnification using the parameters outlined in table B of S1 File. Further information on screen set-up and reagents can be found in S1 File, Supplemental Methods.

### SAβGal assay

Transfections were performed as described in S1 File. 5 days after transfection cells were fixed with 0.5% glutaraldehyde (pH 7.2)(Agar Scientific), washed with PBS containing 1mM MgCl_2_ solution and stained with 1 mg/mL X-gal solution diluted in β-galactosidase solution (0.12mM potassium ferricyanide [K_3_Fe(CN)_6_] and 0.12 mM potassium ferrocyanide [K_4_Fe(CN)_6_] in PBS containing 1mM MgCl_2_ solution, adjusted to pH7 (A375P melanoma cells) or pH6 (all other cell types) using 0.1M citric acid) overnight at 37°C in an incubator without CO_2._ The assay was terminated by washing 3x in PBS when clear positively stained cells could be detected in the etoposide control wells. Cells were counterstained with 0.1μg/ml DAPI dilactate at room temperature and stored at 4°C protected from light. Images were captured on the ArrayScan (Thermo Fisher Scientific) using the Brightfield Module and parameters outlined in table B of S1 File to detect cytoplasmic SAβGal staining. All chemicals were from Sigma Aldrich unless stated otherwise.

### 2D and 3D proliferation assays using DLD1 isogenic lines

DLD1 parental and KRAS^G13D/-^ cells were grown in McCoy’s medium with 10% FBS (Gibco). For the 2D component, cells were seeded at 3000 cells per well in 96 well plates and incubated at 37°C in 5% CO_2_. Cell proliferation was quantified using Alamar Blue (10% v/v; Invitrogen) after 72 hours. For the 3D component, plates were coated with a 0.6% agar–medium mix and allowed to solidify. Cells were suspended in a semisolid 0.4% agar–medium mix, plated at 3000 cells per well and topped with 0.6% agar–medium. Cell proliferation was assessed by Alamar Blue (10% v/v) after 7 days.

### siRNA proliferation assays in 2D and 3D

siGenome SMARTpool reagents (Dharmacon) were reconstituted and diluted to give a final assay concentration of 25 nM in tissue culture treated (2D) or low attachment (Corning, 3D) 96 well plates. These were allowed to complex with Lipofectamine RNAimax (ThermoFisher) as per the manufacturer’s instructions. DLD1 cells were diluted to a density of 3000 cells per well, layered on to the siRNA–lipid mix and incubated to allow reverse transfection to take place. Plates were then topped up with either medium (2D) or agar–medium mix (3D) and cell proliferation quantified using Alamar Blue (10% v/v) after 72h (2D) or 7 days (3D).

### Caspase 3/7 assays

To determine caspase 3/7 levels, the Promega (Southampton, UK) Apo-ONE homogenous caspase 3/7 assay was performed, according to the manufacturer’s instructions. Transfections were performed as described in S1 File. 5 days post-transfection, cells were harvested according to the assay instructions. 100μl per well of caspase 3/7 reagent was added to each well and incubated for 4 hours prior to plate read using Safire II plate reader (Tecan Trading AG, Switzerland).

### Profiling inflammatory markers

Inflammatory markers including several known components of the SASP were analysed using the Mesoscale Discovery (Rockville, USA) human 9-plex pro-inflammatory tissue culture kit (K15007B). Transfections were performed as described in S1 File. Tissue culture supernatants were obtained at 5 days post-transfection. Calibrators and controls were prepared according to the manufacturer’s instructions. 25μl supernatants were incubated for 2 hours at room temperature with shaking in assay plates pre-coated with multiplex capture antibody body cocktails. Detection antibodies were prepared according to the manufacturer’s instructions and 25μl per well of 1x detection antibodies were added to assay plates for 2 hours at room temperature with shaking. After binding of detection antibodies, plates were washed 3 times with the supplied wash buffer and read buffer T added. Plates were analysed immediately on Mesoscale Discovery Quickplex SQ120 plate reader. Hits were taken to be genes for which at least 2/3 siRNA caused induction of cytokines by at least mean + 3SD of scrambled control transfections.

### Microarray processing and analysis

RNA was labelled and amplified using the one-colour microarray gene expression analysis protocol (Agilent Technologies, Santa Clara, CA), hybridised to Agilent whole human genome 4 x 44k Agilent whole human genome microarrays and incubated for 17h at 60°C in a hybridisation oven. Arrays were washed on a magnetic stirrer using Agilent wash buffers. Slides were scanned on an Agilent microarray scanner at 5μm resolution, PMT at 100% and 10%. The extended dynamic range setting was corrected for saturation. Microarray data were extracted using Agilent Feature Extraction software (Agilent Technologies, Santa Clara, CA). All array data were analysed in GeneSpring for normalisation and statistical analysis (Agilent Technologies, Santa Clara, CA). Intra-array normalisation was carried out using the 75^th^ percentile for each microarray. Significant differences in expression between scrambled and ECT2 transfected cells were determined in both parental and *KRAS*^+/-^ HCT116 cells using unpaired t-test. One array was prepared for each of the ECT2 siRNAs against 5 samples from scrambled control transfectants in parental cells. In *KRAS*^+/-^ cells, 2 scrambled control samples were used. IDs with p<0.03 were selected for further analysis. Microarray data are available in the Gene Expression Omnibus with accession number GSE100459.

### MetaCore network analysis

Differentially expressed genes were analysed using the shortest paths algorithm in MetaCore (Thomson Reuters) with ECT2 as a seed object. Individual networks were initially built for differentially expressed genes in HCT116 parental and *KRAS*^+/-^ backgrounds. These were merged to generate Fig 7.

## Supporting information

S1 Fig A large-scale morphology screen identifies siRNAs that induce a senescent-like morphology in A375P melanoma cells. (a) Scatterplot showing results of a rescreen of 810 siRNAs representing all 3 siRNA oligos included in the Ambion druggable genome library for the top 270 gene hits in A375P. (b) Scatterplot showing the position of all 3 siRNAs targeting the top 40 genes taken forward to validate the senescence response in secondary assays. Robust hits had > 2 siRNAs passing the cut-off. Graphs drawn in Tableau desktop represent mean nuclear area per well against mean number of objects (nuclei) per well for each siRNA. Cut-off values mean scrambled control– 1 sd for number of objects and mean scrambled + 1, 2 or 3 sd for nuclear area are shown. Orange crosses mark test siRNAs passing the cut-off for nuclear area increase, while green crosses mark test siRNAs below the cut-off. Etoposide 10μM are shown as blue circles, CDK1 siRNA positive controls shown as blue addition sign, scrambled siRNA negative controls are shown as blue squares. Etoposide control is excluded from (b) for clarity.
(TIF)

S2 Fig Expression of senescence biomarkers allows validation and refinement of screen hits. Validation of the top 40 hit siRNAs for nuclear area increase in A375P (a) and HCT116 (b) ranked in descending order. Mean nuclear area per well (μm^2^) for triplicate wells in 3 independent transfections is represented as box whisker plots generated in Tableau desktop. Boxes represent the 25^th^– 75^th^ percentile of the data. Median level is shown as a colour change within the box. Positive (CDK1) and negative (Scrambled) siRNA controls are shown. Mean SAβGal expression in A375P (c) and HCT116 (d). Graphs drawn in Microsoft Excel represent the mean and standard error of triplicate wells from 3 independent transfections expressed as a fold change of scrambled control.
(TIF)

S3 Fig Expression of p21 and 53BP1 associated with senescence inducing siRNAs. Representative images of p21 (top) and 53BP1 (bottom) staining for siRNA hits and scrambled controls in A375P (a) and HCT116 (b).
(TIF)

S4 Fig Caspase 3/7 activity and ECT2 levels after ECT2 knockdown in *KRAS* mutant HCT116 parental cells. (a) Promega Apo-ONE caspase 3/7 assay in HCT116 parental cells. Cells were left untransfected (cells) or were transfected with ECT2, CDK1, or scrambled siRNA. Cell culture medium was included as negative control. Assays were performed 5 days post-transfection. After addition of assay reagent, cells were incubated for 4h prior to plate read. Mean + SEM of 2 independent experiments in triplicate. (b) Expression levels of ECT2 following knockdown. Microarrays were prepared and processed as described in materials and methods. Mean intensities of ECT2 probes in cell RNA preparations corresponding to transfection with 3 independent siRNAs were compared with 5 replicate scrambled transfections. Mean + SEM shown relative to scrambled.
(TIF)

S5 Fig Set-up of a morphology based screen for siRNAs inducing a senescent phenotype. (a) Screen overview. A375P melanoma cells were transfected with 50nM siRNA from the Ambion Druggable Genome Library. 5 days later cells were fixed, stained for DAPI and imaged using the Operetta high content imaging platform. Nuclei in acquired images were detected and quantified using Harmony software and the output exported to excel for further analysis of hits. (b) Representative DAPI images of controls included on all screen plates, etoposide compound control for senescent morphology, CDK1 siRNA positive control and scrambled siRNA non-targeting control. (c) Representative analysis of cell proliferation in screen controls determined by a count of mean number of objects (DAPI stained nuclei). Cut-off was set to mean scrambled control -1sd for proliferation (red line). (d) Representative analysis of nuclear area increase in screen controls determined by mean nuclear area per well (μm^2^). Cut-off was set to mean scrambled control +1sd for nuclear area (red line). (e) Representative images of SAβGal stained cells 5 days after transfection with siRNA targeting CDK1 (left) or scrambled (right). Scale bar represents 100 μm.
(TIF)

S1 File Supporting methods, figure legends, and tables. Supporting methods include details of cell lines and culture methods, siRNA transfection methods, screen set-up, statistical analyses and antibodies. Supporting table A shows Operetta analysis sequences and algorithms used. Supporting table B shows parameters used for ArrayScan analysis.
(DOCX)

S2 File Validation of the cellular senescence screen. This file provides details of assays performed using etoposide and a kinase inhibitor library to determine optimal phenotypic parameters for use in the screen. Senescence was initially evaluated in A375P cells by a range of assays including growth, colony formation, SAβGal, p21 and 53BP1, H2AX and nuclear area. From the results of the validation, the primary screen was subsequently performed as described in the text.
(DOCX)
