# Supplementary figures and images for: A ‘synthetic-sickness’ screen for senescence re-engagement targets in mutant cancer backgrounds
Source: PLoS Genet. 2017 Aug 14;13(8):e1006942. doi: 10.1371/journal.pgen.1006942 (PMC5570495; doi:10.1371/journal.pgen.1006942)

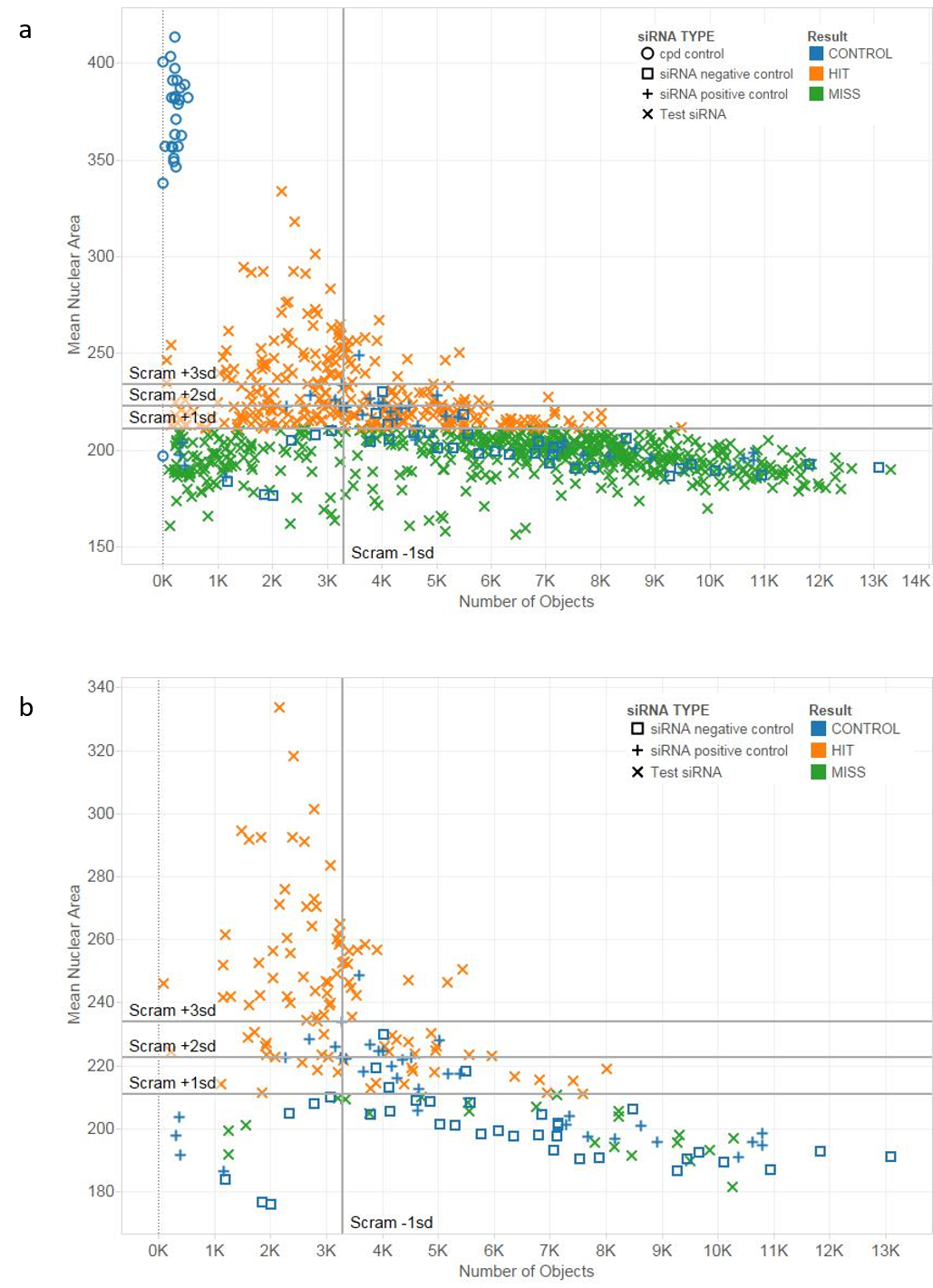

Supplement: S1 Fig — (a) Scatterplot showing results of a rescreen of 810 siRNAs representing all 3 siRNA oligos included in the Ambion druggable genome library for the top 270 gene hits in A375P. (b) Scatterplot showing the position of all 3 siRNAs targeting the top 40 genes taken forward to validate the senescence response in secondary assays. Robust hits had > 2 siRNAs passing the cut-off. Graphs drawn in Tableau desktop represent mean nuclear area per well against mean number of objects (nuclei) per well for each siRNA. Cut-off values mean scrambled control– 1 sd for number of objects and mean scrambled + 1, 2 or 3 sd for nuclear area are shown. Orange crosses mark test siRNAs passing the cut-off for nuclear area increase, while green crosses mark test siRNAs below the cut-off. Etoposide 10μM are shown as blue circles, CDK1 siRNA positive controls shown as blue addition sign, scrambled siRNA negative controls are shown as blue squares. Etoposide control is excluded from (b) for clarity. (TIF) [file pgen.1006942.s001.tif]

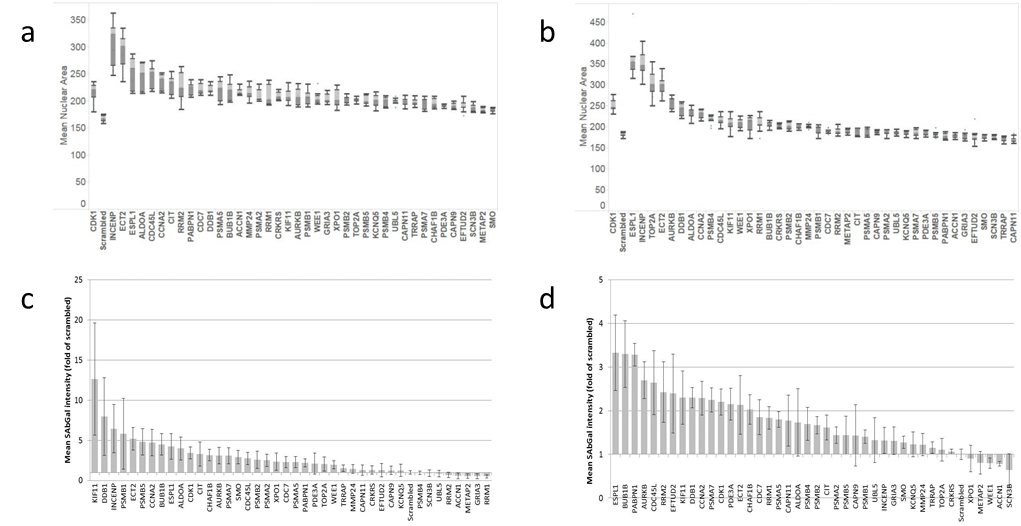

Supplement: S2 Fig — Validation of the top 40 hit siRNAs for nuclear area increase in A375P (a) and HCT116 (b) ranked in descending order. Mean nuclear area per well (μm2) for triplicate wells in 3 independent transfections is represented as box whisker plots generated in Tableau desktop. Boxes represent the 25th– 75th percentile of the data. Median level is shown as a colour change within the box. Positive (CDK1) and negative (Scrambled) siRNA controls are shown. Mean SAβGal expression in A375P (c) and HCT116 (d). Graphs drawn in Microsoft Excel represent the mean and standard error of triplicate wells from 3 independent transfections expressed as a fold change of scrambled control. (TIF) [file pgen.1006942.s002.tif]

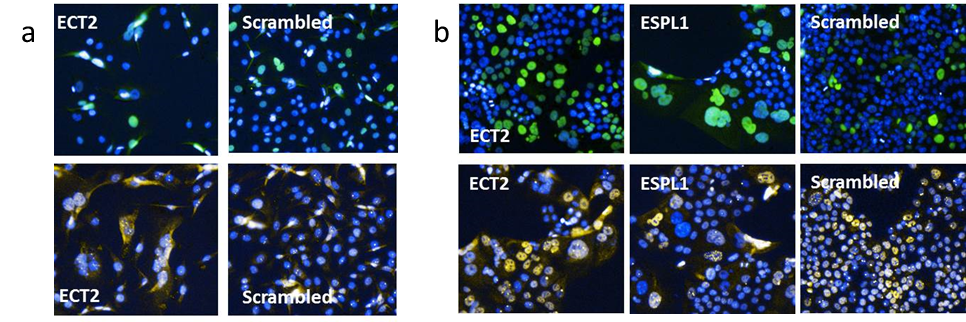

Supplement: S3 Fig — Representative images of p21 (top) and 53BP1 (bottom) staining for siRNA hits and scrambled controls in A375P (a) and HCT116 (b). (TIF) [file pgen.1006942.s003.tif]

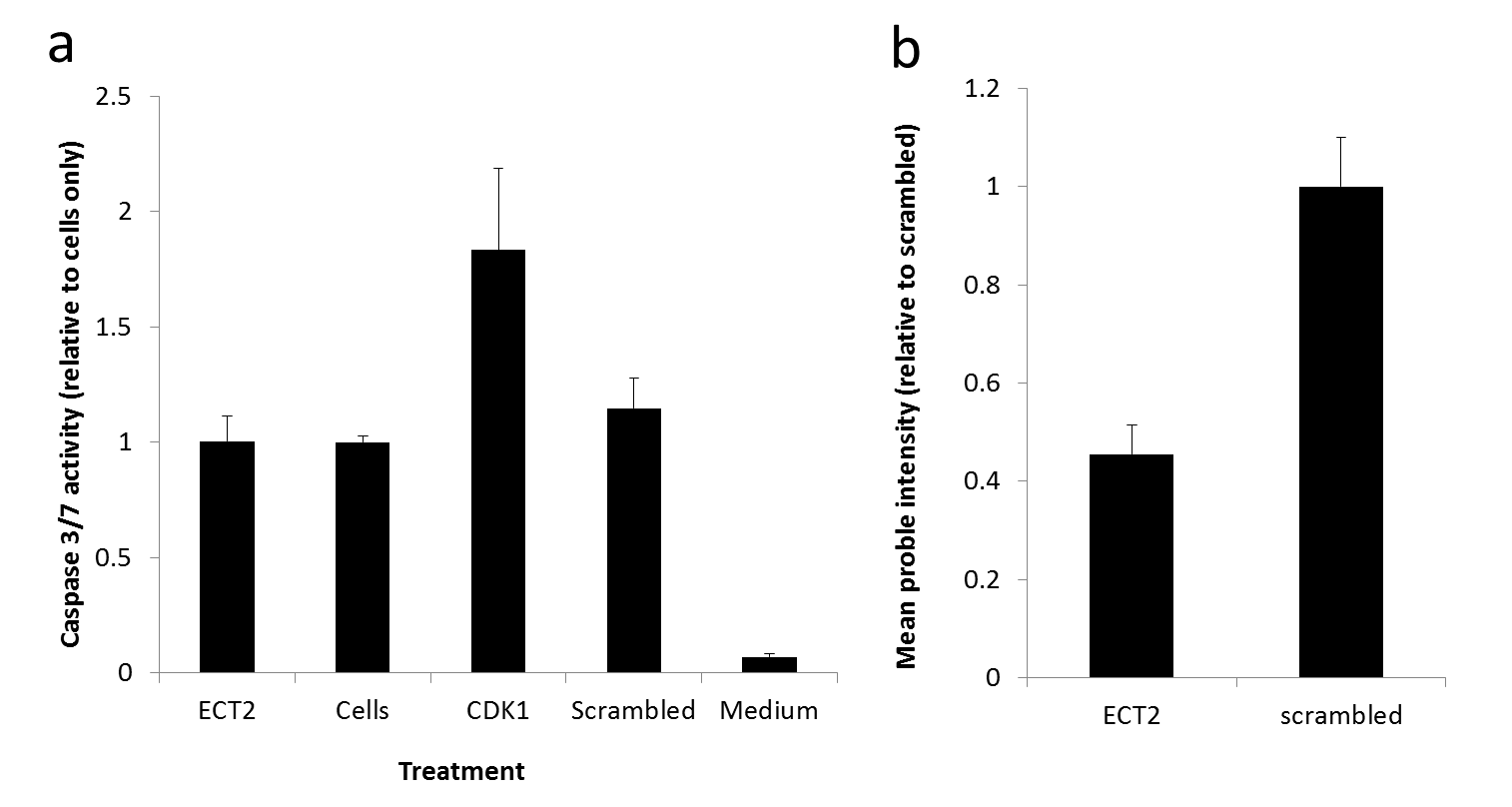

Supplement: S4 Fig — (a) Promega Apo-ONE caspase 3/7 assay in HCT116 parental cells. Cells were left untransfected (cells) or were transfected with ECT2, CDK1, or scrambled siRNA. Cell culture medium was included as negative control. Assays were performed 5 days post-transfection. After addition of assay reagent, cells were incubated for 4h prior to plate read. Mean + SEM of 2 independent experiments in triplicate. (b) Expression levels of ECT2 following knockdown. Microarrays were prepared and processed as described in materials and methods. Mean intensities of ECT2 probes in cell RNA preparations corresponding to transfection with 3 independent siRNAs were compared with 5 replicate scrambled transfections. Mean + SEM shown relative to scrambled. (TIF) [file pgen.1006942.s004.tif]

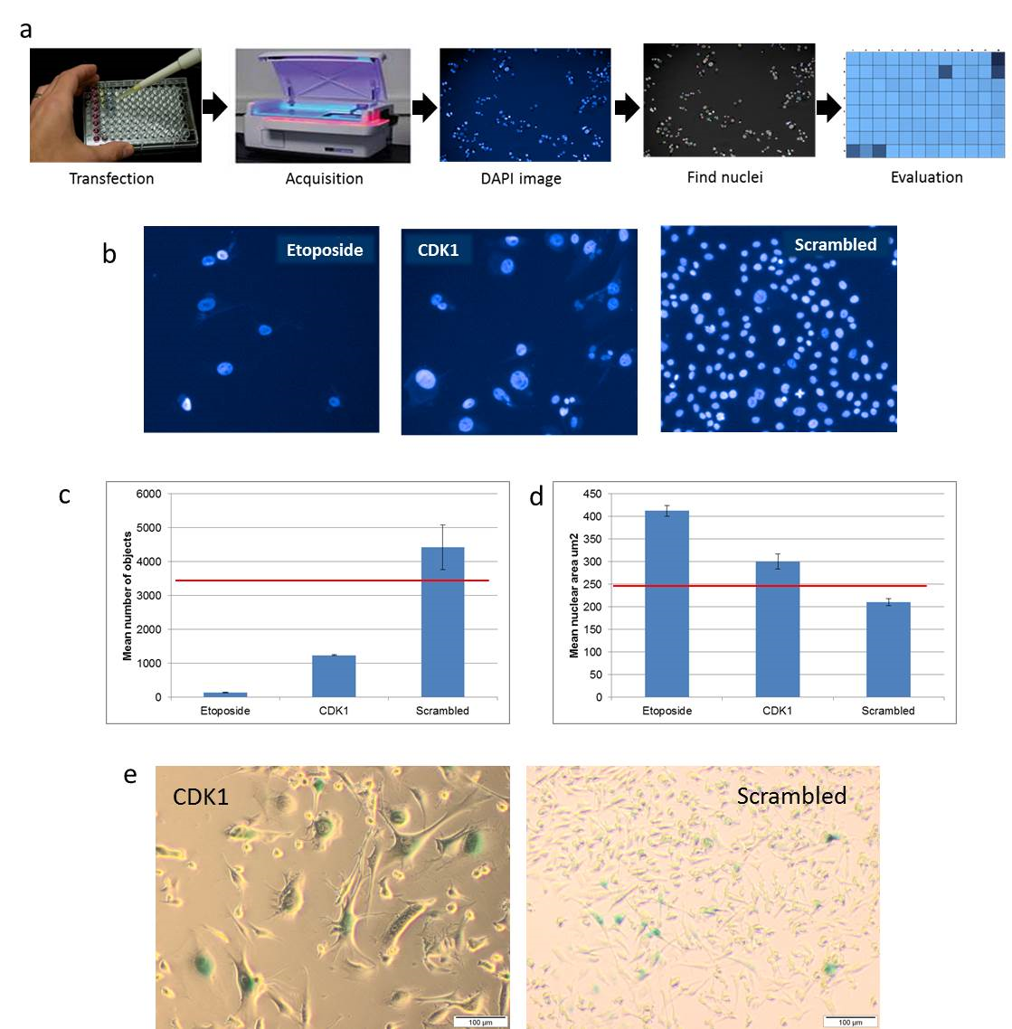

Supplement: S5 Fig — (a) Screen overview. A375P melanoma cells were transfected with 50nM siRNA from the Ambion Druggable Genome Library. 5 days later cells were fixed, stained for DAPI and imaged using the Operetta high content imaging platform. Nuclei in acquired images were detected and quantified using Harmony software and the output exported to excel for further analysis of hits. (b) Representative DAPI images of controls included on all screen plates, etoposide compound control for senescent morphology, CDK1 siRNA positive control and scrambled siRNA non-targeting control. (c) Representative analysis of cell proliferation in screen controls determined by a count of mean number of objects (DAPI stained nuclei). Cut-off was set to mean scrambled control -1sd for proliferation (red line). (d) Representative analysis of nuclear area increase in screen controls determined by mean nuclear area per well (μm2). Cut-off was set to mean scrambled control +1sd for nuclear area (red line). (e) Representative images of SAβGal stained cells 5 days after transfection with siRNA targeting CDK1 (left) or scrambled (right). Scale bar represents 100 μm. (TIF) [file pgen.1006942.s005.tif]
