# Supplementary material for: A ‘synthetic-sickness’ screen for senescence re-engagement targets in mutant cancer backgrounds
Source: PLoS Genet. 2017 Aug 14;13(8):e1006942. doi: 10.1371/journal.pgen.1006942 (PMC5570495; doi:10.1371/journal.pgen.1006942)
Supplement: S1 File — Supporting methods include details of cell lines and culture methods, siRNA transfection methods, screen set-up, statistical analyses and antibodies. Supporting table A shows Operetta analysis sequences and algorithms used. Supporting table B shows parameters used for ArrayScan analysis. (DOCX) [file pgen.1006942.s006.docx]

**Supporting methods**

**Cell lines and culture**

The A375P melanoma cell line was cultured as previously described ([40](#_ENREF_40)). A375P cells were batch frozen and freshly thawed for batches of 14 screen plates to ensure that the primary screen, rescreen and subsequent validation assays were performed on cells of the same passage. All other cell lines were obtained from Horizon Discovery plc. HCT116 and DLD1 colorectal carcinoma isogenic cell lines were maintained in McCoys 5a medium (Thermo Fisher Scientific) containing 10% FBS and 2mM glutamine. MCF10a parental cells were maintained in DMEM-F12 (Thermo Fisher Scientific) supplemented with 5% horse serum (PAA), 2mM L-glutamine, 0.1µg/ml cholera toxin (Sigma Aldrich), 20ng/ml hEGF, 10µg/ml insulin (Sigma Aldrich) and 0.5µg/ml hydrocortisone (Sigma Aldrich) and MCF10a KRAS^G13D/+^ were maintained in DMEM-F12 supplemented with 2% charcoal-dextran treated FBS (PAA), 2mM L-glutamine, 0.1µg/ml cholera toxin, 20ng/ml hEGF, 10µg/ml insulin and 0.5µg/ml hydrocortisone.

**siRNA transfection and reagents**

The Ambion Silencer Select Druggable Genome siRNA library and all reordered hit siRNAs were obtained from Applied Biosystems and resuspended to 5μM in nuclease-free water prior to first use. For downstream validation assays reordered hit siRNAs and controls were aliquoted into 96-well master plates from which all assays were performed. Transfection conditions were optimised using non-targeting Silencer Select negative control siRNA #2 (Ambion, 4390846) and CDK1 (CDC2 siRNA gene solution, Qiagen, 1027416 gene ID 983) siRNA to give a seeding density that allowed detection of individual nuclei by Harmony image analysis software (PerkinElmer) at endpoint, lipofectamine concentration that produced the least toxicity and experiment duration that allowed detection of a robust senescence response in the shortest time to prevent outgrowth of untransfected cells. A z’ score of 1> Z ≥ 0.5 calculated from mean nuclear area per well for non-targeting negative and CDK1 positive control siRNAs confirmed assay optimisation ([41](#_ENREF_41)). For all assays transfection of siRNA was carried out by reverse transfection in 96-well format using 50nM siRNA and 0.3µl lipofectamine 2000 (Thermo Fisher Scientific) in Nunc 96-well black optical bottom tissue culture plates (Thermo Fisher Scientific). Triplicate wells transfected with 50nM non-targeting negative control and 50nM CDK1 positive control siRNAs were included on each plate as a baseline from which to define hit cut-offs and quality control respectively. In addition duplicate wells treated with 10µM etoposide (Sigma Aldrich) were included as a robust positive control for detection of senescent morphology.

**Screen set-up**

Our initial screen was performed in the A375P melanoma cell line, which has the ability to senesce, displays known biomarkers of senescence, is easily transfected and is amenable to high content screening. Screen parameters were optimised in A375P using siRNA targeting CDK1 (cyclin dependent kinase 1), which was subsequently used as the positive control siRNA in all assays. Knock-down of CDK1 has previously been shown to be associated with a senescence response ([14](#_ENREF_14)) and in our hands showed biomarkers of senescence in transient transfection and gave a robust morphological senescence-like response that was easily distinguished from the non-targeting siRNA control. Treatment with 10μM etoposide was also included as a control for senescent nuclear morphology, although it is known to induce overt toxicity in the majority of treated cells, with only a minor population undergoing true senescence ([15](#_ENREF_15), [16](#_ENREF_16)) (Supplemental Figure 5). Hit siRNAs were defined as those that induced a large, flattened nuclear morphology and decreased cellular proliferation in a large fraction of the cell population.

**Data Analysis and Statistics**

Text output from image analysis was exported into Microsoft excel for further evaluation and identification of hits. For the primary screen hits were determined by increased nuclear area and decreased proliferation, as measured by number of nuclei, in comparison to controls. Cut-off values of mean nuclear area (μm^2^) plus 1 standard deviation (sd) and mean number of nuclei (objects) minus 1sd calculated across all wells, excluding etoposide as an extreme outlier, were determined for each plate, based on the assumption that most wells would not show a senescent phenotype. siRNAs passing the cut-off at mean +/- 1, 2 and 3 sd were recorded and hits were then ranked by confidence based on the number of siRNAs per gene passing the cut-off, such that genes with 3/3 siRNA oligos in the library with mean +/- 1sd > 2/3 siRNA with mean +/- 1sd > 1/3 siRNA with mean +/- 3sd.

Cut-off for the rescreen was a single value determined from the mean nuclear area plus 1sd calculated from the combined mean of triplicate non-targeting siRNA control wells across all rescreen plates, based on the assumption that the majority of wells in the rescreen plates would show a senescence phenotype. Hits were recorded and ranked by confidence as described for the primary screen.

Cut-off for validation assays was defined on a plate-by-plate basis determined by the mean of triplicate non-targeting control wells plus 3sd for nuclear area, p21, 53BP1 and SAβGal. Each test siRNA was transfected in triplicate for all assays and the results of 3 independent experiments were combined. Hits were defined as those that passed the cut-off in at least 2/3 wells in at least 2/3 independent experiments.

**Antibodies and reagents**

For analysis of mean nuclear area and mean number of cells per well cell nuclei were stained with 0.1µg/ml DAPI dilactate (Thermo Fisher Scientific) in Dulbecco’s phosphate buffered saline (PBS) containing 0.2% Triton X-100. Antibodies used for immunofluorescence of senescence biomarkers were as follows: Mouse monoclonal [EA10] to p21 (Abcam, Ab16767), Rabbit polyclonal to 53BP1 (Cell Signalling, 4937), Alexa Fluor 488 goat-anti-mouse (Thermo Fisher Scientific), Alexa Fluor 546 goat-anti-rabbit (Thermo Fisher Scientific).

**Supporting figure legends**

**S1 Fig. A large-scale morphology screen identifies siRNAs that induce a senescent-like morphology in A375P melanoma cells.** (a) Scatterplot showing results of a rescreen of 810 siRNAs representing all 3 siRNA oligos included in the Ambion druggable genome library for the top 270 gene hits in A375P. (b) Scatterplot showing the position of all 3 siRNAs targeting the top 40 genes taken forward to validate the senescence response in secondary assays. Robust hits had > 2 siRNAs passing the cut-off. Graphs drawn in Tableau desktop represent mean nuclear area per well against mean number of objects (nuclei) per well for each siRNA. Cut-off values mean scrambled control – 1 sd for number of objects and mean scrambled + 1, 2 or 3 sd for nuclear area are shown. Orange crosses mark test siRNAs passing the cut-off for nuclear area increase, while green crosses mark test siRNAs below the cut-off. Etoposide 10μM are shown as blue circles, CDK1 siRNA positive controls shown as blue addition sign, scrambled siRNA negative controls are shown as blue squares. Etoposide control is excluded from (b) for clarity.

**S2 Fig. Expression of senescence biomarkers allows validation and refinement of screen hits.** Validation of the top 40 hit siRNAs for nuclear area increase in A375P (a) and HCT116 (b) ranked in descending order. Mean nuclear area per well (µm^2^) for triplicate wells in 3 independent transfections is represented as box whisker plots generated in Tableau desktop. Boxes represent the 25^th^ – 75^th^ percentile of the data. Median level is shown as a colour change within the box. Positive (CDK1) and negative (Scrambled) siRNA controls are shown. Mean SAβGal expression in A375P (c) and HCT116 (d). Graphs drawn in Microsoft Excel represent the mean and standard error of triplicate wells from 3 independent transfections expressed as a fold change of scrambled control.

**S3 Fig. Expression of p21 and 53BP1 associated with senescence inducing siRNAs.** Representative images of p21 (top) and 53BP1 (bottom) staining for siRNA hits and scrambled controls in A375P (a) and HCT116 (b).

**S4 Fig. Caspase 3/7 activity and ECT2 levels after ECT2 knockdown in *KRAS* mutant HCT116 parental cells**

(a) Promega Apo-ONE caspase 3/7 assay in HCT116 parental cells. Cells were left untransfected (cells) or were transfected with ECT2, CDK1, or scrambled siRNA. Cell culture medium was included as negative control. Assays were performed 5 days post-transfection. After addition of assay reagent, cells were incubated for 4h prior to plate read. Mean + SEM of 2 independent experiments in triplicate. (b) Expression levels of ECT2 following knockdown. Microarrays were prepared and processed as described in materials and methods. Mean intensities of ECT2 probes in cell RNA preparations corresponding to transfection with 3 independent siRNAs were compared with 5 replicate scrambled transfections. Mean + SEM shown relative to scrambled.

**S5 Fig. Set-up of a morphology based screen for siRNAs inducing a senescent phenotype.** (a) Screen overview. A375P melanoma cells were transfected with 50nM siRNA from the Ambion Druggable Genome Library. 5 days later cells were fixed, stained for DAPI and imaged using the Operetta high content imaging platform. Nuclei in acquired images were detected and quantified using Harmony software and the output exported to excel for further analysis of hits. (b) Representative DAPI images of controls included on all screen plates, etoposide compound control for senescent morphology, CDK1 siRNA positive control and scrambled siRNA non-targeting control. (c) Representative analysis of cell proliferation in screen controls determined by a count of mean number of objects (DAPI stained nuclei). Cut-off was set to mean scrambled control -1sd for proliferation (red line). (d) Representative analysis of nuclear area increase in screen controls determined by mean nuclear area per well (μm^2^). Cut-off was set to mean scrambled control +1sd for nuclear area (red line). (e) Representative images of SAβGal stained cells 5 days after transfection with siRNA targeting CDK1 (left) or scrambled (right). Scale bar represents 100 μm.

**Supporting Tables**

Table A: Operetta Analysis Sequences

| **Assay** | Algorithm | Method | Channel | Output for analysis |
| --- | --- | --- | --- | --- |
| Nuclear area | Find nuclei | B | DAPI | Area (μm^2^) Mean |
| p21 expression | Find nuclei  Calculate intensity properties | B  Standard | DAPI  488 | 488 in nucleus Mean |
| 53BP1 expression | Find nuclei  Find spots | B  A | DAPI  546 | Spots per nucleus Mean |

Table B: ArrayScan Analysis

| **Assay** | **ArrayScan channels** | **ArrayScan assay for analysis** | **Primary output features selected for analysis** |
| --- | --- | --- | --- |
| SA-β galactosidase | Ch1 = **DAPI**  Ch2 (brightfield) = **SA-β galactosidase** | SpotDetector.V4 | SA-β galactosidase expression: **MEAN_ObjectSpotTotalIntenCh2/ SpotTotalIntenPerObjectCh2** |
| Immunofluorescence (nuclear area, 53BP1 foci and p21 expression) | Ch1 = **DAPI**  Ch2 = **53BP1 foci**  Ch3 = **p21** | CompartmentalAnalysis.V4 | Nuclear area =  **MEAN_ObjectAreaCh1**  53BP1 nuclear foci: **MEAN_CircSpotCountCh2**  p21 nuclear intensity: **MEAN_CircTotalIntenCh3** |
