# Supplementary material for: A ‘synthetic-sickness’ screen for senescence re-engagement targets in mutant cancer backgrounds
Source: PLoS Genet. 2017 Aug 14;13(8):e1006942. doi: 10.1371/journal.pgen.1006942 (PMC5570495; doi:10.1371/journal.pgen.1006942)
Supplement: S2 File — This file provides details of assays performed using etoposide and a kinase inhibitor library to determine optimal phenotypic parameters for use in the screen. Senescence was initially evaluated in A375P cells by a range of assays including growth, colony formation, SAβGal, p21 and 53BP1, H2AX and nuclear area. From the results of the validation, the primary screen was subsequently performed as described in the text. (DOCX) [file pgen.1006942.s007.docx]

**Validation of the screen for cellular senescence**

**Validation of etoposide as positive inducer of cell senescence**

**
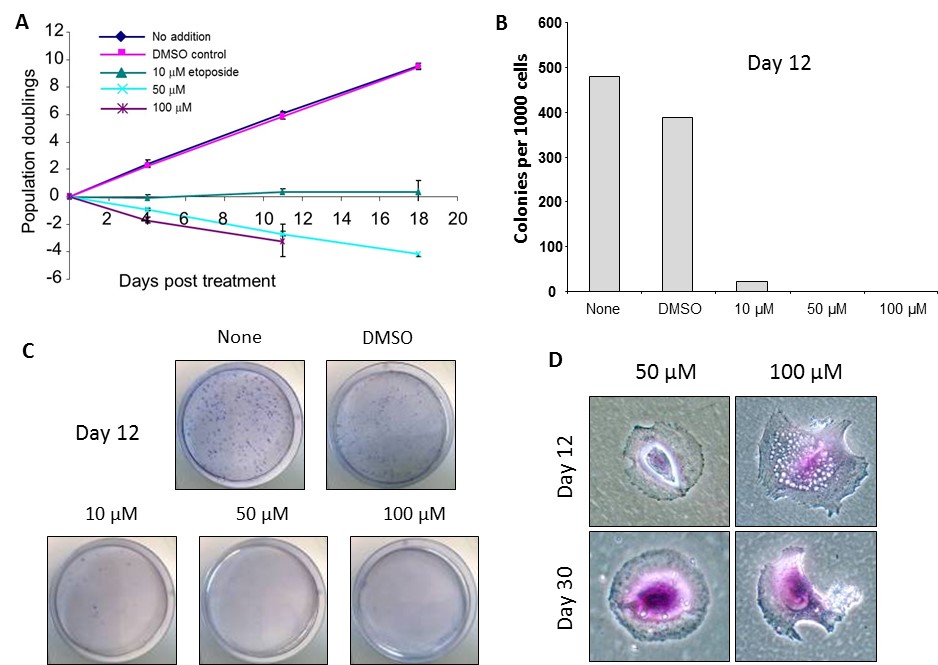
**Initially etoposide (10 µM, added for 48 hr) was investigated and established as a positive control agent to induce cell senescence in the A375P melanoma cell line (Figure 1). The A375P cell line was used as a validation line as its senescence response is well characterised. Cell population growth curves showed that cell number was static after this treatment, followed by culture without the agent (Figure 1A). Colony-formation assays showed that very few of the cells were now able to proliferate and form colonies (Figure 1B, C). Colony formation was completely lost at 50 and 100 µM etoposide; these concentrations also gave some reduction in cell number (toxicity) (Figure 1A). At all these concentrations, surviving single nondividing cells could be seen which were large and flat, as expected for senescent cells (Figure 1D).

**Figure 1. Induction of permanent arrest in cells by etoposide.** Typical experiments shown. A375P cells were plated at 3 x 10^4^ cells/ml and grown with or without etoposide at the stated concentrations or DMSO (vehicle control) for 48 h (in triplicate) before the following assays. (A) Growth curves. Cells were harvested, counted by hemocytometer and replated without drug at 3 x 10^4^ cells/ml, then counted and subcultured at the times shown. Counts were converted to cumulative population doublings. Means and SEM are shown. Cell number was static up to 18 days following exposure to etoposide at 10 µM. (B,C). Colony-formation potential. Cells were plated at 100 cells/ml (1000 per 10-ml plate) and grown for a further 12 days. Plates were fixed and stained with crystal violet before colonies were counted (B) or photographed (C). Only about 3% of cells regrew. (D) Large, flat, single cells survived up to 12 days or (in other experiments) 30 days later, even with higher etoposide concentrations.

**
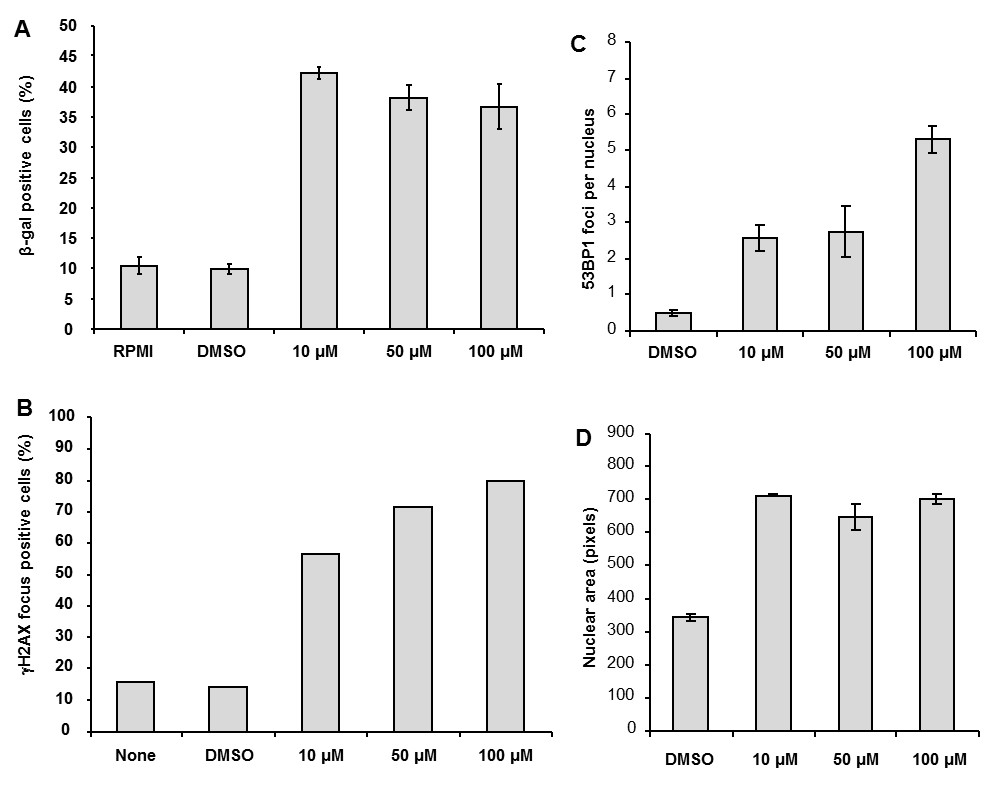
**A375P cells following 48 h culture with 10 µM etoposide and 7 days without compound were then tested for molecular markers of cell senescence, and showed substantial levels of all tested markers. Figure 2 shows induction of SAβgal and persistent foci of γH2AX and 53BP1. Marker positivity was below 100%, at least in part because a small percentage of melanoma cells did escape and proliferate (Figure 1B), forming a disproportionate fraction of the cells after a week.

**Figure 2. Induction of molecular senescence markers and increased nuclear area in cells by etoposide.** A375P cells were grown with etoposide, vehicle control or no addition (“RPMI” or “none”) for 48 h, then for 7 days with two medium changes, without additions. Cells were then fixed and used for β-galactosidase assays (A), immunostaining (B,C) or DAPI staining (D), and image analysis (C,D). Means and SEM are shown. β-galactosidase assays were at pH 7.0 since melanocytes and melanoma cells can be positive at pH 6.0 even when growing. γH2AX is another marker of DNA-damage foci, like 53BP1, and immunostained cells were counted by eye. 53BP1 foci and nuclear area were quantitated by automated image analysis [20].

Mean nuclear area was also substantially increased, being approximately doubled with etoposide concentrations of 10 µM or more (Figure 2D). p21 was also efficiently induced (Figure 3) (tested after 5 days’ continuous treatment here, in comparison to siRNAs as in main text). Toxicity was high after 5 days with 10 µM etoposide, but most surviving cells expressed p21. Thus a state resembling normal senescence could be induced by a drug in malignant melanoma cells, and the characteristics included increased nuclear area and persistent cell cycle arrest.

**
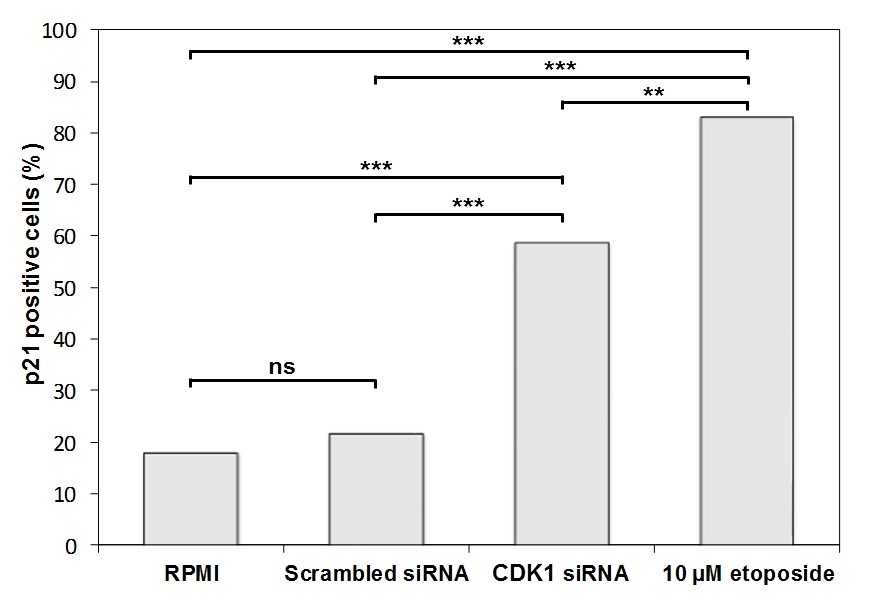
Figure 3. Induction of p21 by etoposide.** A375P cells were grown with etoposide (10 µM) or the siRNAs shown for 5 days continuously. Culture details, p21 immunostaining and automated counting were as described in the main text (Materials and Methods). Asterisks represent significance testing by 1-way ANOVA, Newman-Keuls Multiple Comparison Test (**p<0.01, ***p<0.001). p21 was induced in over 80% of surviving cells by etoposide, although cell death was also seen with this longer exposure.

**Efficacy of cell-based screen for A375P cells, using kinase inhibitor library as an example**

Our cell-based senescence screening of a kinase inhibitor library (InhibitorSelect; Calbiochem/Merck) in IMR90 human diploid fibroblasts was described previously [20]. The primary screen criteria were increased mean nuclear area and decreased number of nuclei (cells) following fixation and DAPI staining. Compounds scoring highly in this screen were frequently able to induce cell senescence as assessed by loss of colony-forming potential and upregulation of a number of senescence markers including p53, p21, SAβgal, HMGA2, hypophosphorylated RB1 and others [20].

We screened the same library of 160 compounds in the same way on A375P cells. Thirty-five compounds rated as initial hits in the automated screen, of which 8 were also hits in IMR90 cells. Seven of these 8 compounds produced highly significant reductions in colony forming potential in A375P cells, of which three gave large reductions: RHO-associated kinase (ROCK1) inhibitor IV, JNK inhibitor IX, and SU6656, inhibitor of SRC tyrosine kinase family members (Figure 4A). Aloisine A, an inhibitor of CDK1, CDK2, CDK5, GSK3α, and JNK, inhibited colony formation by around 50%. Inspection of the colony assay plates showed single, large cells surviving in each case, confirming arrest (Figure 4D). All these four were further tested in other secondary assays at varying concentrations. The first three compounds gave large increases in nuclear area in the tested concentration range, comparable to that with etoposide, while aloisine A did not affect nuclear area (Figure 4B). ROCK Inhibitor IV and especially JNK inhibitor IX gave increases in 53BP1 focus formation (Figure 4C), and both were good inducers of SAβgal, even at 3 µM (Figure 4E).

In conclusion, the primary automated screen for nuclear size and number was able to identify compounds or treatments that could induce senescence in melanoma cells, in combination with secondary screening for molecular markers.

**
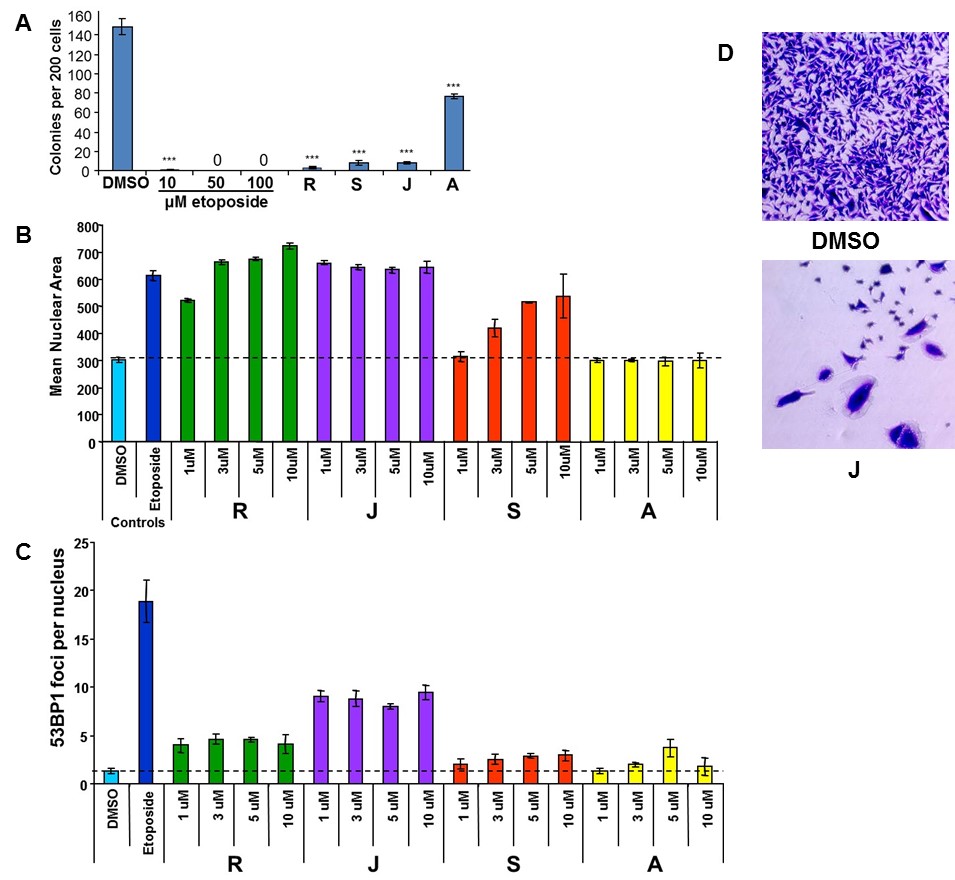
**


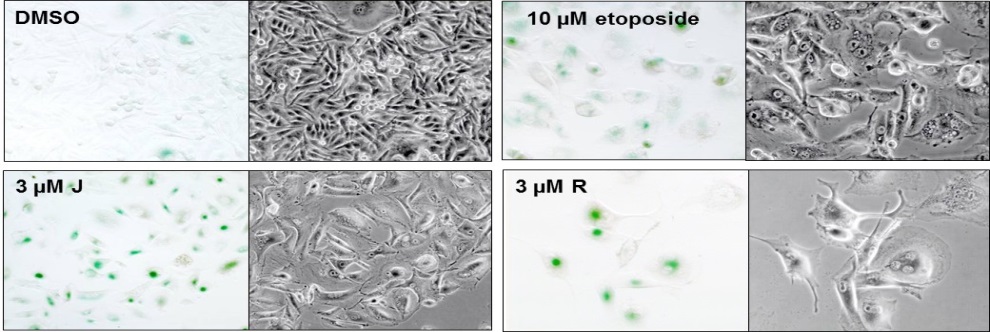


**E**

**Figure 4. Induction of senescence markers in A375P cells by 3/4 compounds that scored highly in the primary screen of a kinase inhibitor library.** Compounds: (R) ROCK1 inhibitor IV. (J) JNK inhibitor IX. (S): SU6656. (A): aloisine A. Cells were grown with the compounds for 48 h and then without for 7 d, or 12 d for colony assays. Panels: (A) All these inhibitors at 10 µM reduced colony formation (measured as in Figure 1 of this file) highly significantly. Typical large surviving cells on a colony plate are shown in panel (D), with some growing cells (colony) for comparison. Shown for JNK inhibitor IV as an example. (B) Aloisine A did not increase nuclear area at these concentrations, while the other compounds did. (C) ROCK inhibitor IV and JNK inhibitor IX both increased 53BP1 focus formation at all tested concentrations. (D) See panel (A). (E) Efficient induction of SAβgal by JNK inhibitor IX and ROCK inhibitor IV. Means and SEM shown throughout.
